# The Current State of Research on Sirtuin-Mediated Autophagy in Cardiovascular Diseases

**DOI:** 10.3390/jcdd10090382

**Published:** 2023-09-06

**Authors:** Yuqin Wang, Yongnan Li, Hong Ding, Dan Li, Wanxi Shen, Xiaowei Zhang

**Affiliations:** 1 The Second Clinical Medical College, Lanzhou University, Lanzhou 730106, China; wangyq21@lzu.edu.cn (Y.W.);; 2 Department of Cardiac Surgery, Lanzhou University Second Hospital, Lanzhou 730031, China; lyngyq2006@foxmail.com; 3 Department of Cardiology, Lanzhou University Second Hospital, Lanzhou 730031, China; dingh0110@163.com; 4 Qinghai Provincial People’s Hospital, Qinghai University, Xining 810007, China

**Keywords:** Sirtuins, autophagy, FOXOs, AMPK, mTOR, cardiovascular diseases, Sirtuins-based therapies

## Abstract

Sirtuins belong to the class III histone deacetylases and possess nicotinamide adenine dinucleotide-dependent deacetylase activity. They are involved in the regulation of multiple signaling pathways implicated in cardiovascular diseases. Autophagy is a crucial adaptive cellular response to stress stimuli. Mounting evidence suggests a strong correlation between Sirtuins and autophagy, potentially involving cross-regulation and crosstalk. Sirtuin-mediated autophagy plays a crucial regulatory role in some cardiovascular diseases, including atherosclerosis, ischemia/reperfusion injury, hypertension, heart failure, diabetic cardiomyopathy, and drug-induced myocardial damage. In this context, we summarize the research advancements pertaining to various Sirtuins involved in autophagy and the molecular mechanisms regulating autophagy. We also elucidate the biological function of Sirtuins across diverse cardiovascular diseases and further discuss the development of novel drugs that regulate Sirtuin-mediated autophagy.

## 1. Introduction

In 1987, SIR2 was first discovered as a transcriptional silencer in yeast cells by Rine et al. [1]. Subsequently, seven SIR2 homologs, namely SIRT1–SIRT7, were identified. SIRT2 is primarily localized in the cytoplasm, whereas SIRT1, SIRT6, and SIRT7 are predominantly localized in the nucleus, and SIRT3, SIRT4, and SIRT5 are primarily situated within the mitochondria. Rapid advancements in the field of proteomics have enabled researchers to elucidate that the subcellular localization of Sirtuin proteins is contingent upon the specific cell type, cellular state, and molecular interactions. For example, SIRT1 and SIRT2 shuttle between the nucleus and cytoplasm, interacting with proteins present in both cellular compartments, and SIRT3 is also expressed in the cytoplasm [2]. Under physiological conditions, mammalian Sirtuins exhibit enzymatic functions that translate and modify various histone and non-histone proteins, thereby inducing or inhibiting the expression of downstream target proteins, which participate in multiple physiological processes such as glucose metabolism, fatty acid metabolism, insulin secretion, ATP synthesis, DNA repair, and cell cycle regulation. Upon exposure to endogenous or exogenous stimuli, Sirtuins participate in pathological processes such as oxidative stress, autophagy, apoptosis, and inflammatory responses [3].

Autophagy is not only a crucial process during cardiac development but also an adaptive cellular response to starvation, hypoxia, metabolic irregularities, oxidative stress, aging, the accumulation of aberrant proteins and organelles, and other external stimuli [4]. In circumstances of normal or mild stress, autophagy maintains cellular energy homeostasis by degrading and recycling intracellular waste and damaged organelles. However, under severe stress, increased activation of autophagy leads to non-selective degradation of normal mitochondria and mitochondrial-related proteins, thereby exacerbating mitochondrial damage, affecting energy metabolism, and causing energy imbalance [5]. Autophagy occurs in various types of cardiovascular cells, including cardiomyocytes, vascular smooth muscle cells, fibroblasts, macrophages, and endothelial cells [6]. Cardiomyocytes are terminally differentiated cells and rely considerably on autophagy for the elimination of abnormal substances [7]. Therefore, the regulation of autophagy is crucial for the maintenance of cardiovascular homeostasis.

Both Sirtuins and autophagy are acknowledged as crucial factors in the pursuit of prolonging lifespan and protecting organisms against age-related diseases and metabolic disorders [8,9,10,11]. Sirtuin-mediated autophagy is activated or suppressed under pathological conditions and plays a vital role in some cardiovascular diseases, including atherosclerosis (AS), myocardial ischemia/reperfusion (MI/R) injury, hypertension, heart failure, diabetic cardiomyopathy (DCM), drug-induced myocardial damage, and cardiogenesis/cardiac maintenance [11,12,13,14,15,16,17,18,19,20,21]. Sirtuins exert their influence on autophagy by regulating the gene expression of autophagy-related proteins and their post-translational modifications, thereby affecting their activity and subcellular localization [10]. Undoubtedly, a more comprehensive understanding of the cross-regulation between Sirtuins and autophagy may provide crucial insights and novel therapeutic avenues for addressing cardiovascular diseases. However, there is currently a dearth of comprehensive literature providing a concise overview of the role of Sirtuins in the regulation of autophagy in cardiovascular diseases. This review underscores the key molecular mechanisms through which Sirtuins regulate cardiovascular autophagy, shedding light on the biological roles of Sirtuins across diverse cardiovascular diseases. In addition, we summarize novel medical therapeutic strategies targeting Sirtuin-mediated autophagy.

## 2. Regulation of Cardiovascular Autophagy by Various Sirtuins

### 2.1. Sirtuins in the Nucleus

**Figure 1:**
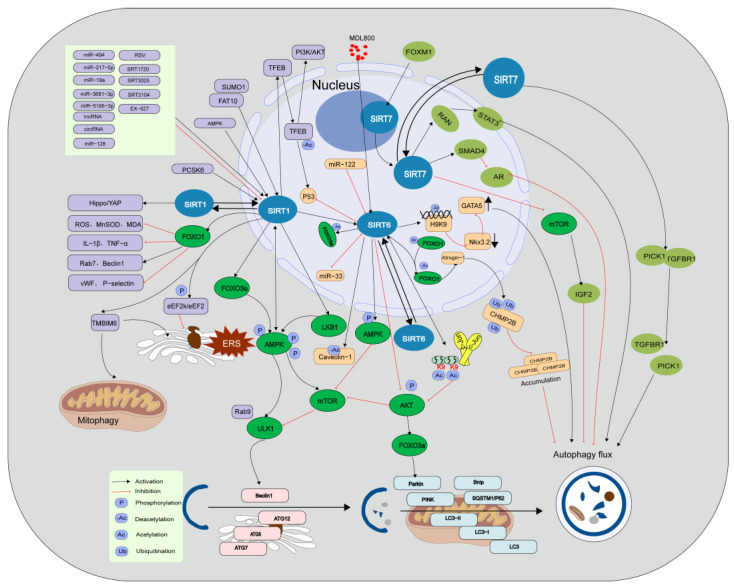
Role of nuclear Sirtuins in the regulation of cardiovascular autophagy. AKT, protein kinase B; AMPK, adenosine 5′-monophosphate (AMP)-activated protein kinase; AR, androgen receptor; ATG12, autophagy-related 12; ATG5, autophagy-related 5; ATG7, autophagy-related 7; Bnip, BCL2 interacting protein; CHMP2B, CHarged Multivesicular body Protein 2B; circRNA, circular RNA; DOX, doxorubicin; eEF2, eukaryotic elongation factor 2; eEF2k, eukaryotic elongation factor 2 kinase; ERS, endoplasmic reticulum stress; EX-527, SIRT1 inhibitor; FAT10, human leukocyte antigen- F adjacent transcript 10; FOXM1, the forkhead box M1; FOXO1, the forkhead box 1; FOXO3a, the forkhead box O3a; GATA5, GATA-binding protein 5; GTSP1, glutathione S-transferase P1; IGF2, insulin-like growth factor 2; IL-1B, interleukin-1B; LncRNA, Long noncoding RNA; LC3, microtubule-associated protein light chain 3; LKB1, liver kinase B1; MDA, malondialdehyde; MDL800, SIRT6 activator; miR-122, microRNA 122; miR-128, microRNA 128; miR-19a, microRNA 19a; miR-217-5p, microRNA 217-5p; miR-33, microRNA 33; miR-3681-3p, microRNA 3681-3p; miR-494, microRNA 494; miR-5195-3p, microRNA 5195-3p; MnSOD2, manganese superoxide dismutase 2; mTOR, mammalian target of rapamycin; Nkx3.2, NK3 homeobox 2; PARK2, parkin RBR E3 ubiquitin protein ligase; PCSK6, proprotein convertase subtilisin/kexin type 6; PI3K, phosphoinositide 3--kinase; PICK1, protein interacting with C kinase 1; PINK, PTEN induced putative kinase; RSV, Resveratrol; SIRTs, silent information regulators; SMAD4, mothers against decapentaplegic homolog 4; SQSTM1, Sequestosome1; STAT3, signal transducer and activator of transcription 3; SUMO1, small ubiquitin−like modifier 1; TFEB, transcription factor EB; TGFBR1, Type1 transforming growth factor beta receptor; TNF-α, tumor necrosing factor alpha; ULK1, Unc-51-like autophagy activating kinase 1; Vwf, von Willebrand factor; YAP, yes-associated protein.

SIRT1, a recognized regulator of autophagy, induces autophagy directly by deacetylating autophagy-related genes or increases autophagic flux by upregulating the expression of autophagy-regulating genes [22]. Studies have shown that adenosine monophosphate-activated protein kinase (AMPK) and various non-coding RNAs regulate the participation of SIRT1 in autophagy in cardiovascular cells. Under conditions of restricted energy, such as glucose limitation, serum starvation, amino acid deprivation, and MI/R, AMPK phosphorylation leads to the activation of SIRT1, which in turn enhances the transcription of autophagy-related genes [14]. AMPK can also be activated as a downstream molecule of SIRT1. For example, SIRT1 directly phosphorylates AMPK to activate the unc-51-like autophagy activating kinase 1 (ULK1) pathway or inhibit the participation of mammalian target of rapamycin (mTOR) in autophagy. SIRT1 also directly deacetylates forkhead box O3a (FOXO3a) to activate AMPK and autophagy [23,24,25]. Furthermore, SIRT1-mediated regulation of autophagy also involves non-coding RNAs, such as microRNAs (miRNAs), long non-coding RNAs (lncRNAs), and circular RNAs (circRNAs). For example, miRNA-494 targets SIRT1 through the phosphoinositide 3-kinase (PI3K)/protein kinase B (AKT)/mTOR signaling pathway to inhibit autophagy in myocardial cells [26]; miR-128 inhibits the PIK3R1/AKT/mTORC1 and/or SIRT1/p53 pathways to exacerbate angiotensin II (Ang II)-induced pathological autophagy [27]. miR-217-5p and miR-19a downregulate SIRT1 expression to regulate autophagy and exert cellular protective effects [28,29]. CircRNAs regulate SIRT1 at the transcriptional and post-translational levels through miR-3681-3p and miR-5195-3p sponging. In vitro experiments have shown that silencing SIRT1 restores the effect of the upregulation of circ-SIRT1 expression on Ang II-induced autophagy [30]. LncRNAs enhance oxidative modification of low-density lipoprotein (ox-LDL)-induced macrophage autophagy through the SIRT1/mitogen-activated protein/nuclear factor kappa-B pathway [31]. SIRT1 positively regulates autophagy in cardiovascular diseases. For example, SIRT1 promotes autophagy by deacetylating autophagy marker proteins such as forkhead box-1 (FOXO1) and Beclin1 [22,32]. SIRT1 activates downstream effectors of peroxisome proliferator-activated receptor γ coactivator 1-α (PGC-1α) and fibroblast growth factor 21 (FGF21) to promote autophagy [33]. Additionally, SIRT1 promotes transcription factor EB (TFEB) nuclear translocation and deacetylation, thereby activating the p53 and PI3K/AKT signaling pathways to promote autophagy and maintain mitochondrial dynamics balance [19,34]. During endoplasmic reticulum stress (ERS), SIRT1 expression is suppressed, and autophagy decreases. Conversely, inhibiting SIRT1 attenuates ERS-induced autophagy, and activating SIRT1 enhances autophagy protection against ERS-induced cell death through ERS pathways [35]. In addition, mitochondrial autophagy-associated proteins, such as PTEN-induced putative kinase (PINK1), autophagy-related 5 (ATG5), microtubule-associated protein light chain 3 (LC3), and Beclin1, are downregulated in cardiomyocytes stimulated by hypoxia/reoxygenation (H/R) injury. However, activating the SIRT1/transmembrane BAX inhibitor motif containing 6 (TMBIM6) signaling pathway improves mitochondrial autophagy and ERS [36]. Human leukocyte antigen-F adjacent transcript 10 (FAT10) affects autophagy by regulating SIRT1 degradation, decreasing the nuclear translocation of SIRT1, and inhibiting its activity through its C-terminal glycine residue. FAT10 competes with small ubiquitin-like modifier 1 (SUMO1) for SIRT1’s K734 modification site, thereby further reducing LC3 deacetylation and ultimately inhibiting autophagy [37]. Hippo/Yes-associated protein (YAP) is one of the primary signaling pathways that responds to various mechanical stimuli and mediates inflammation. Unidirectional laminar flow of blood induces endothelial protection, whereas a disruption in blood flow results in a pro-atherosclerotic response. When blood flow is disrupted, endothelial cells exhibit reduced phosphorylation of YAP at the Ser127 residue, which results in an increase in YAP expression and its nuclear translocation and the suppression of autophagy. Laminar flow of blood increases SIRT1 expression to inhibit YAP activity and promote YAP nuclear translocation. Additionally, SIRT1 overexpression reduces the expression of downstream genes of YAP, such as *CTGF* and *CYR61*, in situations where blood flow is disrupted, whereas inhibition of SIRT1 expression upregulates *CTGF* and *CYR61* activity [38]. In monocytes, inhibiting SIRT1 activates mTOR, which in turn inhibits autophagy, induces inflammation, and promotes the progression of AS [39] (Figure 1).

SIRT6 plays a crucial role in the regulation of cellular autophagy by primarily inhibiting AKT and activating AMPK to deacetylate autophagy-associated proteins. For example, SIRT6 deficiency promotes H3K9 acetylation and c-Jun promoter binding as well as its transcriptional activity to suppress AKT signaling, thereby inhibiting mTOR activation and promoting autophagy [40]. SIRT6 promotes FOXO3-dependent autophagy by reducing AKT protein levels and phosphorylation, thereby promoting the formation of LC3-II and downregulating p62 expression [41]. The upstream miR-122 inhibits SIRT6, thereby exacerbating the decrease in Ang II stimulation-induced autophagic flux, which in turn leads to increased cell migration, oxidative stress, and apoptosis [42]. SIRT6 deacetylates caveolin-1 to trigger its autophagic degradation, whereas knocking out SIRT6 induces autophagy. SIRT6 regulates cardiac autophagy through the FOXO3-dependent pathway and activates autophagy induction factors, such as ATG5 and lysosome-associated membrane protein-2, while inhibiting autophagy suppressors, such as p53 and mTOR [43]. Activated SIRT6 reduces acetylation of FOXO1, thereby promoting the transcriptional function of atrogin-1, which ultimately promotes atrogin-1-mediated degradation of charged multivesicular body protein 2B [44]. SIRT6 deacetylates histone H3K9 to inhibit NK3 homeobox 2 transcription, thereby promoting autophagy and preventing endothelial injury [45] (Figure 1).

SIRT7, a Sirtuin primarily localized in the nucleolus, is relatively less studied for its role in autophagy regulation [46,47]. Silencing forkhead box M1 promotes cellular autophagy by regulating the SIRT7/mTOR/insulin-like growth factor 2 pathway [48]. In osteoarthritis, increased SIRT7 expression in chondrocytes activates autophagy to prevent cartilage degeneration [49]. In prostate cancer cells, SIRT7 indirectly promotes autophagy by regulating the androgen receptor signaling pathway, while its depletion substantially inhibits androgen-induced cell autophagy [50]. Additionally, SIRT7 promotes the deacetylation of RNA N-deacetylase by competitively binding with Ribosomal L1 domain-containing protein 1, thereby leading to the accumulation of nuclear signal transducer and activator of transcription 3 (STAT3) and STAT3-regulated autophagy. However, a few studies have demonstrated that SIRT7 promotes the dissociation of proteins that interact with C kinase 1 and the transforming growth factor β receptor in the cytoplasm, which inhibits their autophagic degradation [51]. At present, the role of SIRT7-mediated autophagy in cardiovascular diseases remains unclear; hence, in vivo and in vitro trials must be conducted in the future (Figure 1).

### 2.2. Sirtuins in the Mitochondria

**Figure 2:**
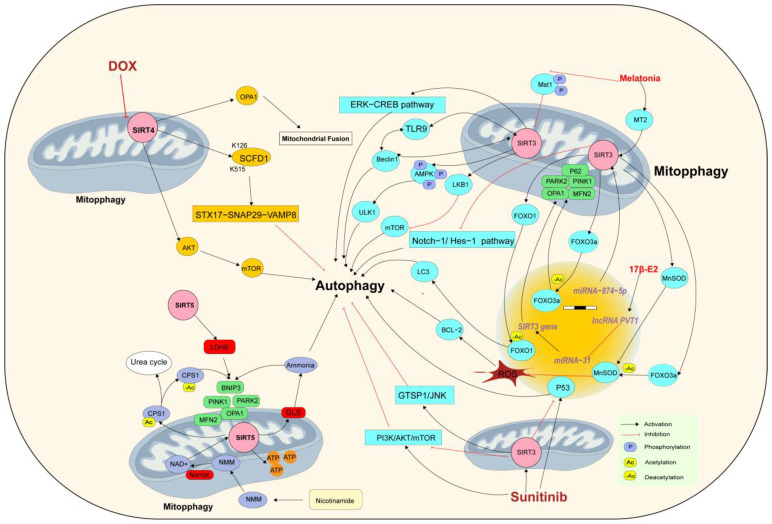
Role of mitochondrial Sirtuins in the regulation of cardiovascular autophagy. 17β-E2,17beta-estradiol; AKT, protein kinase B; AMPK, adenosine 5′-monophosphate (AMP)-activated protein kinase; BCL-2, B cell lymphoma 2; Bnip, BCL2 interacting protein; CPS1, carbamoyl-phosphate synthase 1; CREB, cAMP-response element binding protein; ERK, extracellular regulated kinase; FOXO1, the forkhead box 1; FOXO3a, the forkhead box O3a; GLS, glutaminase; Hes-1, hairy and Enhancer of split homolog-1; IL-1B, Interleukin-1B; LncRNA PVT1, Long noncoding RNA PVT1; LC3, microtubule-associated protein light chain 3; LDHB, lactate dehydrogenase B; LKB1, liver kinase B1; MFN2, mitofusin 2; miRNA-31, microRNA 31; miRNA-874-5p, micro 874-5p; MnSOD2, manganese superoxide dismutase; Mst1, macrophage stimulating 1; MT2, melatonin membrane receptor 2; mTOR, mammalian target of rapamycin; NAD^+^, nicotinamide adenine dinucleotide; Nampt, nicotinamide phosphoribosyl transferase; OPA1, optic atrophy 1; PARK2, parkin RBR E3 ubiquitin protein ligase; ROS, reactive oxygen species; SCFD1, sec1 family domain containing 1; ULK1, unc-51-like autophagy activating kinase 1.

SIRT3 plays a dual role in the regulation of autophagy in cardiovascular disease, which can not only enhance but also attenuate autophagy. SIRT3 primarily regulates cellular energy metabolism homeostasis by modulating mitochondrial autophagy [13,52]. For example, SIRT3 deacetylates liver kinase B1 (LKB1) and manganese superoxide dismutase 2 (MnSOD2), leading to AMPK phosphorylation. Phosphorylated AMPK then directly activates ULK1, thereby inducing Beclin1-mediated macro-autophagy while inhibiting mTOR-dependent autophagy [13,53]. Additionally, SIRT3 directly inhibits the PI3K/AKT/mTOR pathway or activates p53 to induce autophagy and prevent drug-induced cardiotoxicity in tumors [54]. SIRT3 suppresses excessive activation of mitochondrial autophagy by deacetylating FOXO3a and MnSOD2 [55,56]. Moreover, SIRT3 overexpression exacerbates drug-induced cell death by inhibiting the SIRT3/glutathione S-transferase P1(GSTP1)/c-Jun N-terminal kinase (JNK) autophagy pathway [57]. SIRT3 upregulates the expression of autophagy-related proteins, namely Beclin1 and LC3-II, by downregulating the Notch-1/Hes-1 pathway [58]. Under conditions of a high-fat diet, the expression of SIRT3 is decreased, which leads to the inactivation of the extracellular regulated kinase (ERK)/cAMP-response element binding protein (CREB) pathway, ultimately resulting in the inhibition of Bnip3-mediated mitochondrial autophagy. SIRT3 overexpression restores Bnip3 expression and mitochondrial autophagy while suppressing the inhibitory effect of the ERK/CREB axis on SIRT3 activation and the enhancement of mitochondrial autophagy [59]. In individuals with diabetes, SIRT3 upregulates mitochondrial autophagy in the heart solely by inhibiting macrophage stimulating 1 (Mst1), and inhibiting SIRT3 expression impairs mitochondrial autophagy in myocardial cells, thereby exacerbating type 1 DCM [60]. In human umbilical vein endothelial cells (HUVECs), the upregulation of lncRNA PVT1 expression by 17beta-estradiol inhibits miR-31, activates the SIRT3 promoter, and upregulates SIRT3 expression, thereby promoting autophagy and inhibiting H_2_O_2_-induced HUVEC aging [61,62]. In addition, the regulation of autophagy involves the dynamic communication between the Beclin1-TLR9-SIRT3 complex [63]. In vascular smooth muscles, miRNA-874-5p regulates autophagy by targeting SIRT3 [64] (Figure 2).

SIRT4 is localized in the mitochondria as well as the cytoplasm and exhibits the catalytic capabilities of various enzymes, allowing it to participate in various pathological and physiological processes, such as energy metabolism, oxidative stress, autophagy, and aging. Studies have revealed that SIRT4 interacts with the optic atrophy 1 protein to promote mitochondrial fusion, inhibit mitochondrial autophagy, regulate mitochondrial quality control, suppress oxidative stress, and delay aging [65]. SIRT4 overexpression inhibits doxorubicin (DOX)-induced cardiac toxicity by suppressing the AKT/mTOR autophagy pathway [66]. Furthermore, research has revealed that SIRT4 deacetylates sec1 family domain containing 1 at the K126 and K515 residues, thereby facilitating the formation of the STX17-SNAP29-VAMP8 complex and promoting autophagy [67]. To date, limited studies have been conducted on the involvement of SIRT4 in autophagy regulation, particularly in cardiovascular diseases. Further investigations are necessary to elucidate the role of SIRT4 in mitochondrial autophagy (Figure 2).

To date, the research on SIRT5-mediated autophagy has primarily focused on the regulation of the urea cycle, and investigations in relation to cardiovascular diseases are limited. A few studies suggest that SIRT5 modulates ammonia levels by deacetylating carbamoyl phosphate synthetase 1 in the mitochondria, thereby regulating mitochondrial autophagy and dynamics [68]. SIRT5 converts glutamine to glutamate and ammonia by desuccinylating glutaminase in the mitochondria, increasing mitochondrial reactive oxygen species (ROS), and inducing mitochondrial autophagy [69]. Another study suggested that SIRT5 increases lactate dehydrogenase B activity in the cytoplasm by deacetylating it at the K329 residue, thus promoting autophagy [70] (Figure 2).

### 2.3. Sirtuins in the Cytoplasm

**Figure 3:**
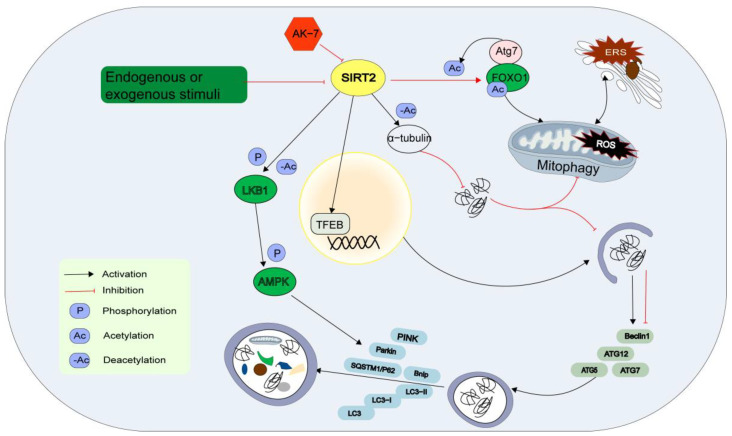
Role of cytoplasmic Sirtuins in the regulation of cardiovascular autophagy. AK-7, SIRT2 inhibitor; AMPK, adenosine 5′-monophosphate (AMP)-activated protein kinase; ATG5, autophagy -related 5; ATG7, autophagy-related 7; ATG12, autophagy-related 12; BNIP3, BCL2 interacting protein 3; ERS, endoplasmic reticulum stress; FOXO1, the forkhead box 1; LC3, microtubule-associated protein light chain 3; LKB1, liver kinase B1; PARK2, parkin RBR E3 ubiquitin protein ligase; ROS, reactive oxygen species; SQSTM1, sequestosome1; TFEB, transcription factor EB.

The precise mechanism by which SIRT2 regulates cardiovascular autophagy remains unclear. Starvation, oxidative stress, and cold exposure decreased SIRT2 expression, resulting in an increase in FOXO1 acetylation. Acetylated FOXO1 binds to ATG7, promoting its acetylation and inducing ERS and autophagic interactions [71]. SIRT2 directly binds to the 3′-untranslated region of TFEB and enhances its mRNA stability, thereby facilitating autophagosome formation and autophagy component release [72]. In hypertrophic hearts, SIRT2 restores AMPK activity by interacting with LKB1, an upstream kinase of AMPK. Moreover, SIRT2 promotes LKB1 phosphorylation and the subsequent amplification of the LKB1/AMPK signal by binding to LKB1 and deacetylating it at lysine residues [73,74]. SIRT2 deficiency results in increased acetylation of mitochondrial proteins, and its activation is associated with the deacetylation of α-tubulin, which leads to impaired autophagy clearance, impeded transportation, and the elimination of misfolded proteins. However, the loss of SIRT2 function as a result of knocking out the specific inhibitor AK-7 or SIRT2 leads to the restoration of microtubule stability and enhancement of autophagy [75]. Nevertheless, research has validated that SIRT2 is localized not only in the cytoplasm but also within mitochondria, playing a role in autophagy and possibly having unique mitochondrial targets [76] (Figure 3).

## 3. Role of Sirtuin-Induced Autophagy in Cardiovascular Diseases

### 3.1. Atherosclerosis

**Figure 4:**
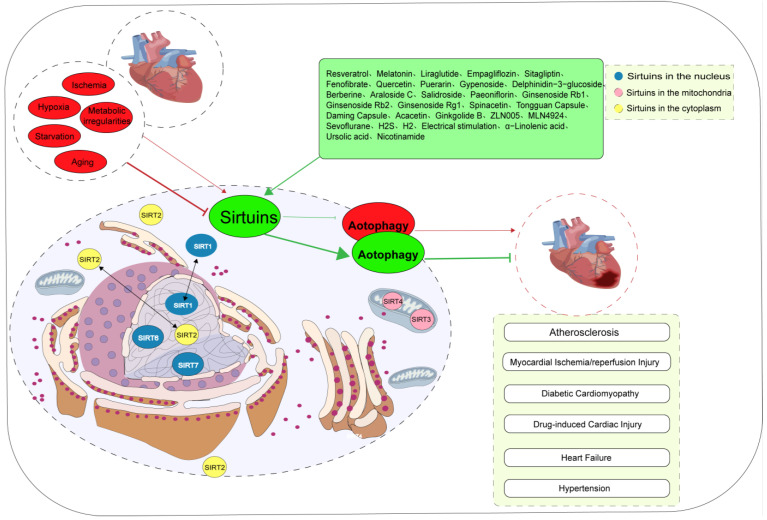
Biological significance of Sirtuin-mediated autophagy in cardiovascular diseases. The subcellular localization of Sirtuins proteins is contingent upon the specific cell type, cellular state, and molecular interactions. Most of the adverse factors, such as ischemia, hypoxia, starvation, metabolic abnormalities, and aging, lead to cardiovascular diseases by inhibiting Sirtuin-mediated autophagy. However, in a few cases, adverse factors contribute to the occurrence of cardiovascular disease by activating Sirtuin-mediated autophagy. At present, a variety of drugs can alleviate various stress injuries by regulating Sirtuin-mediated autophagy and having cardiovascular protective effects. SIRTs are silent information regulators.

AS is a chronic inflammatory disease characterized by elevated levels of LDL cholesterol in the plasma, endothelial dysfunction, inflammation, and immune cell infiltration. Sirtuins have been reported to directly affect AS formation and plaque stability by regulating cellular autophagy to prevent endothelial dysfunction, vascular smooth muscle cell senescence, and foam cell formation [12]. Foam cell formation is one of the key processes in the initial development of AS. Studies have elucidated that SIRT1 activation increases the expression of autophagy-associated proteins and the number of autophagosomes, promotes M2 macrophage polarization, reduces foam cell formation, and decreases plaque area and lipid accumulation, thereby delaying the progression of AS. The inhibition of SIRT1 abolishes these protective responses [77,78]. Inadequate or excessive activation of autophagy in endothelial progenitor cells (EPCs) can lead to endothelial dysfunction, while the restoration of autophagy or the inhibition of excessive autophagy in EPCs promotes vascular regeneration and repair, thereby exerting anti-AS effects [79,80]. Li et al. found that SIRT1 activation may delay AS development by inhibiting autophagy in EPCs through the Wnt/β-catenin/glycogen synthase kinase 3beta signaling pathway [81]. The release of thrombosis factors such as von Willebrand factor (vWF) and P-selectin plays a crucial role in AS and arterial thrombosis. The activation of the SIRT1/FOXO1 signaling pathway-mediated autophagy is a promising target for reducing vWF and P-selectin release and preventing AS [82]. Ma et al. found that inducing SIRT3/FOXO3a pathway-mediated mitophagy results in reduced plaque size and vulnerability, which ultimately alleviates inflammatory responses in AS [83]. Furthermore, SIRT6 overexpression significantly reduces foam cell formation by inducing autophagy in a macrophage foam cell model. Silencing of the key autophagy initiation gene *ATG5* reverses the pro-autophagic effect of SIRT6, resulting in increased foam cell formation. Under conditions of ox-LDL, SIRT6 inhibits the expression of miR-33 and promotes autophagy and cholesterol efflux, thereby reducing foam cell formation in macrophages and attenuating the progression of AS. In contrast, knocking out the *SIRT6* gene aggravates the formation of foam cells and the development of AS [84]. SIRT6 overexpression inhibits the expression of cell adhesion molecules in ox-LDL-treated mouse macrophages, leading to reduced macrophage and foam cell infiltration, significantly increasing macrophage autophagy flux, and thereby inhibiting macrophage apoptosis [85]. SIRT6-mediated autophagy is inhibited in endothelial cells treated with ox-LDL and high concentrations of glucose, whereas SIRT6 overexpression alleviates endothelial cell inflammation and reverses LDL endocytosis [43,86]. High shear stress leads to red blood cell destruction and iron deposition. Elevated iron levels in macrophages in plaques are associated with AS. The activation of SIRT1-mediated autophagy can inhibit the inflammatory response in excess iron autophagy foam cells with excess iron [38,87] (Figure 4).

### 3.2. Myocardial Ischemia/Reperfusion Injury

MI/R injury is a common pathophysiological process in cardiovascular diseases that often leads to reduced myocardium function, non-reflow phenomena, reperfusion arrhythmias, heart failure, and other problems. Factors leading to potential MI/R injury include free radical damage, calcium overload, inflammatory responses, oxidative stress, autophagy, apoptosis, ferroptosis and pyroptosis [88,89,90,91,92,93,94,95,96]. Presently, Sirtuin-mediated autophagy has been suggested to be involved in the process of the development of MI/R injury. In most cases, the activation of Sirtuin-mediated autophagy safeguards myocardial cells against MI/R injury. For example, the activation of the AMPK/SIRT1/FOXO1 and SIRT1/AMPK signaling pathways promotes autophagy, reduces oxidative stress in myocardial cells, significantly reduces myocardial infarct size, and improves heart function [14,97,98]. In addition, ROS generation is the most important signal mechanism in reperfusion-induced injury [99,100]. SIRT3-activated LKB1/AMPK and FOXO3a pathways in the ischemic phase induce the formation of autophagosomes and the activation of Parkin and PINK1, ultimately triggering mitophagy. However, because of the activation of abundant ROS during the reperfusion phase, SIRT3 downregulates the autophagy process by activating superoxide dismutase and eliminating ROS [13,14]. In vivo and in vitro experiments have shown that MI/R reduces SIRT3 expression and its deacetylase activity, which results in decreased antioxidant capacity and enhanced autophagy, whereas the upregulation of SIRT3 expression or ischemia pretreatment attenuates autophagic cell death, improves mitochondrial quality control, and reduces myocardial microvascular damage [101,102]. Recent studies have revealed that myocardin-related transcription factor A alleviates MI/R injury by inducing autophagy, and this protective effect is mediated by SIRT1-dependent autophagy [98]. In some cases, the activation of Sirtuin-mediated autophagy exacerbates MI/R injury. For instance, remote ischemic preconditioning activates SIRT3/hypoxia-inducible factor 1-α and inhibits autophagy, thereby exerting cardioprotective effects [103]. SIRT3 plays an essential role in refeeding syndrome-related myocardial injury during lipopolysaccharide-induced chronic sepsis in rats, possibly via the regulation of PINK/Parkin-mediated mitochondrial autophagy [104]. Low levels of SIRT6 expression have been found to be associated with increased all-cause mortality and notable adverse cardiovascular events in patients with acute myocardial infarction, and the activation of SIRT6-mediated autophagy has been shown to protect endothelial cells from post-ischemic inflammation [86]. Following acute cardiovascular injury in wild-type mice, SIRT7 expression increases, and SIRT7-deficient mice exhibit increased susceptibility to cardiac rupture following myocardial infarction, delayed blood flow restoration following ischemia, impaired wound healing following skin injury, reduced fibrosis and fibroblast differentiation, and decreased inflammatory cell infiltration in the infarct border zone. Additional studies have revealed that SIRT7 participates in tissue repair processes by regulating autophagy [51]. Nevertheless, a few studies suggest that the activation of Sirtuins may have detrimental effects on myocardial cells. For instance, thrombin exacerbates MI/R injury in myocardial cells by activating the SIRT1-mediated autophagy pathway [105] (Figure 4).

### 3.3. Diabetic Cardiomyopathy

DCM is a myocardial-specific microvascular complication in which autophagy is believed to play a dual role [106,107]. Autophagy in the heart of patients with diabetes is influenced by various factors, including blood glucose levels, obesity, insulin levels, glucose toxicity, lipid toxicity, oxidative stress, and inflammation. The activation of autophagy in the heart differs across distinct types of diabetes. For instance, cardiac autophagy is enhanced in type 1 diabetes mellitus but suppressed in type 2 diabetes mellitus [108]. Several studies have shown that the downregulation of SIRT1/SIRT3 contributes to DCM pathology through autophagy-related mechanisms [17,109,110]. For instance, high glucose levels lead to a decrease in the expression of SIRT1 and autophagy marker proteins, whereas the SIRT1 activator SRT1720 enhances autophagy [17]. Exposure of mice to streptozotocin causes myocardial injury and interstitial fibrosis, with increased apoptosis and mitochondrial damage in myocardial cells. In SIRT3-deficient mice, the effect of streptozotocin is more pronounced, and SIRT3 overexpression prevents mitochondrial damage and myocardial cell apoptosis. Further investigations have revealed that the downregulation of mitochondrial autophagy mediated by the SIRT3/FOXO3a/Parkin signaling pathway is a crucial process in the development of DCM [109]. Additionally, the inhibition of neuraminidase 1 activates AMPKα through LKB1, leading to SIRT3 activation, thereby modulating fibrosis, inflammation, apoptosis, and oxidative stress in cardiac tissue during DCM [110] (Figure 4).

### 3.4. Drug-Induced Cardiac Injury

DOX is an effective anthracycline chemotherapy drug, but its clinical application is limited owing to its cardiac toxicity. Autophagy plays a dual role in DOX-induced cardiac toxicity by inducing autophagy at low concentrations and inhibiting autophagy at high concentrations. Moderate levels of autophagy are crucial for organelle renewal and cell survival, whereas excessive activation of autophagy exacerbates cardiac toxicity. For example, experiments involving both in vivo and in vitro DOX-induced cardiac injury models have validated that acute high-dose DOX treatment suppresses AMPK and ULK1 activity, thereby impairing myocardial cell autophagy, whereas restoration of autophagy alleviates cardiac toxicity [111]. Conversely, autophagy has been shown to contribute to the apoptosis of cardiomyocytes and cardiac toxicity in a chronic DOX-induced cardiac toxicity model [112]. Research has shown that Sirtuins play a critical role in the regulation of autophagy during DOX-induced cardiac toxicity [18]. For instance, the activation of SIRT1 deacetylates TFEB and FOXOs, thereby promoting autophagolysosomal elimination and preventing DOX-induced cardiac toxicity [113]. Additionally, SIRT3 activates the mTOR/ULK1 pathway to inhibit NOD-like receptor family pyrin domain-containing 3 (NLRP3) inflammasome activation, restore mitochondrial autophagic flux, and alleviate cardiac toxicity [114]. Sunitinib, a novel anti-tumor drug, can cause hypertension, left ventricular systolic dysfunction, and myocardial cell death. SIRT3 overexpression inhibits autophagy and exacerbates cardiac toxicity, whereas restoration of autophagy by knocking out SIRT3 reverses the aforementioned cardiac toxicity [57]. Moreover, SIRT4 is closely associated with drug toxicity. For instance, in vivo and in vitro experiments have shown that SIRT4 expression improves cardiac function, reduces myocardial cell apoptosis and autophagy, and alleviates cardiac toxicity. However, the activation of mTOR eliminates the protective effect of SIRT4 overexpression on cardiac toxicity. Additional investigations have revealed that during DOX treatment, SIRT4 overexpression activates the AKT/mTOR signaling pathway, which in turn inhibits autophagy. These findings suggest that Sirtuins may serve as prospective targets for treating cardiac toxicity induced by anti-tumor drugs [66]. Currently, research on the involvement of SIRT5 in DOX-induced cardiac toxicity is limited; nevertheless, it is expected to acquire prominence as a critical field of study in the future in connection to drug-induced cardiac toxicity (Figure 4).

### 3.5. Heart Failure

Myocardial remodeling is a considerable pathological process in the development of chronic heart failure, characterized by myocardial hypertrophy, myocardial fibrosis, and apoptosis of myocardial cells. Multiple studies have suggested a potential link between the dysregulation of autophagy and the progression of myocardial remodeling [115]. The maintenance of normal function in myocardial cells relies heavily on basal levels of autophagy, and impaired autophagy leads to myocardial cell hypertrophy [116]. Insufficient autophagy during heart failure is associated with impaired SIRT1/PGC-1α and AMPK signaling, as well as the activation of the Akt/mTOR pathway. The upregulation of SIRT1, PGC-1α, and AMPK, along with inhibition of the Akt/mTOR pathway, promotes autophagy, diminishes myocardial hypertrophy, and improves heart failure [11,19]. The treatment of cardiac cells with Ang II results in a decrease in the expression of SIRT3 and autophagy-related proteins and an increase in the mRNA levels of atrial natriuretic peptide and B-type natriuretic peptide. The activation of SIRT3 promotes autophagy and alleviates Ang II-induced cardiac cell hypertrophy, whereas the silencing of SIRT3 exacerbates myocardial hypertrophy [117,118]. Omentin1 reduces myocardial hypertrophy by upregulating the SIRT3/FOXO3a signaling pathway, thereby initiating mitochondrial autophagy to maintain mitochondrial dynamic balance [52]. Endothelial-to-mesenchymal transition (EndoMT) is a critical pathological process in cardiac fibrosis. Studies have revealed that SIRT3 regulates autophagy-dependent glycolysis during EndoMT. SIRT3 deficiency reduces autophagy, whereas increased autophagy attenuates EndoMT [119]. Additionally, the positive regulation of autophagy by SIRT6 prevents isoproterenol-induced myocardial hypertrophy, possibly by attenuating Akt signaling and promoting accumulation of the FOXO3 transcription factor in the nucleus [40,41]. SIRT7 is involved in processes such as scar formation, angiogenesis, and inflammation. In cardiac fibroblasts, SIRT7 deficiency attenuates transforming growth factor-beta signaling and activates autophagy. This finding suggests that it plays a role in tissue repair by modulating autophagy [51]. Furthermore, SIRT3-deficient mice exhibit sparse cardiac microvasculature, functional hypoxia, impaired cardiac mitochondrial function, and cardiac fibrosis. SIRT3 overexpression restores angiogenic capacity, improves cardiac function, reduces fibrosis, and enhances PINK/Parkin-mediated mitochondrial autophagy, thereby alleviating mitochondrial dysfunction [120] (Figure 4).

### 3.6. Hypertension

A clinical study revealed a significant correlation between a polymorphism (rs2273773) of the SIRT1 gene and dynamic blood pressure levels in patients with hypertension of the Kazakh ethnic group, indicating a potential link between SIRT1 and blood pressure regulation [121]. Recent research has revealed contradictory roles for Sirtuin-mediated autophagy in the development of hypertension. For instance, exposure of rats to arsenic has been shown to significantly increase systolic blood pressure, impair contraction and relaxation responses in isolated aortas to Potassium chloride, Phenylephrine, and Acetylcholine, and upregulate the expression of SIRT1 and autophagy-related proteins [15], suggesting a negative effect of SIRT1-mediated autophagy on hypertension. However, in spontaneously hypertensive rats, SIRT3 expression is significantly decreased, and autophagy is significantly inhibited [16]. Limited studies have been conducted to investigate Sirtuin-mediated autophagy in hypertension. However, Sirtuins are expected to be candidate targets for the treatment of hypertension in the near future (Figure 4).

### 3.7. Cardiogenesis and Cardiac Maintenance

Cardiogenesis is a complex developmental process involving various overlapping stages of cell fate specification, proliferation, differentiation, and morphogenesis. SIRT1 exhibits significance not only in the early stages of embryogenesis but also in stages of cardiogenesis [122]. Li et al. showed that SIRT1 levels substantially decline in type II skeletal muscles, which notably exhibit marked atrophy. SIRT1 overexpression reduces muscle wastage by blocking the activation of FOXO1 and FOXO3 and by downregulating muscle-specific ubiquitin ligases, namely atrogin-1 and muscle RING-finger protein-1, along with multiple autophagy genes [123]. MiRNAs are involved in several core biological processes, including cardiogenesis, hematopoietic lineage differentiation, and oncogenesis. The expression of miR-199a is enhanced during the differentiation of pluripotent stem cells into endothelial cells. Notably, SIRT1 has been identified as a target of miR-199a [124]. Lin28, an RNA-binding protein, plays a role in the regulation of gene translation and is highly expressed in the early stages of embryogenesis. It is essential for modulating the self-renewal of stem cells. Lin28a can upregulate autophagy, inhibit cell damage, and maintain cell morphology and biological function subsequent to I/R injury by activating SIRT1/SIRT3 [20,21]. Consequently, SIRT1 and SIRT3 are compelling targets for cardiogenesis and cardiac maintenance (Figure 4).

## 4. Advances in Sirtuin/Autophagy-Based Therapies

### 4.1. Resveratrol

Resveratrol (RSV) is a naturally occurring compound that exhibits various physiological and biochemical activities. Multiple studies have demonstrated that RSV exerts critical protective effects in cardiovascular diseases by affecting Sirtuin-mediated autophagy [125]. For instance, RSV has been shown to improve the survival rate of rats with heart failure by activating SIRT1 and SIRT3-mediated autophagy and improving hemodynamics and energetics [126]. RSV upregulates SIRT1 expression, enhances autophagic flux, alleviates endothelial cell inflammation, and promotes ox-LDL degradation, thereby preventing arterial thrombosis and AS [82,127,128]. Additional investigations have elucidated that RSV restores mitochondrial quality control following MI/R injury by activating SIRT1/SIRT3-mediated autophagy pathways and reverses cardiac remodeling following extensive myocardial infarction [129,130]. Studies have shown that RSV increases autophagic flux by activating the SIRT1/FOXO1 signaling pathway both in vivo and in vitro. This may serve as a novel strategy for the treatment of DCM [32]. In addition, Carrizzo et al. suggested that the activation of SIRT1 by RSV can rescue vascular dysfunction and prevent thrombosis in patients with methylenetetrahydrofolate-reductase deficiency [131].These findings indicate that RSV represents a promising approach for the treatment of cardiovascular diseases in the future (Table 1, Figure 4).

### 4.2. Melatonin

Several studies have demonstrated that melatonin exerts a protective effect on cardiovascular diseases by regulating Sirtuin-mediated autophagy. For instance, melatonin has been shown to inhibit the progression of AS by activating mitochondrial autophagy through the SIRT3/FOXO3a pathway and attenuating the activation of the NLRP3 inflammasome [83]. Melatonin induces cellular autophagy through the Mst1/SIRT1 signaling pathway and regulates mitochondrial integrity and biogenesis, thereby alleviating post-myocardial infarction cardiac remodeling and dysfunction [132]. Exposure of rats to arsenic has been observed to significantly increase systolic blood pressure and impair contraction and relaxation responses in isolated aortas; additionally, it upregulates the expression of SIRT1 and autophagy-related proteins. Melatonin, however, protects against arsenic-induced vascular toxicity by inhibiting the SIRT1 autophagy pathway [15]. In addition, treating arsenic-exposed rats with melatonin has been shown to alleviate QT interval prolongation, reverse the effects on glutathione and malondialdehyde levels, reduce oxidative stress, inhibit SIRT3/NRF2-mediated autophagy, and decrease the expression of apoptotic proteins (caspase-3 and Bax/Bcl-2) in the myocardium [133]. Long-term melatonin treatment suppresses the progression of DCM and MIR by reactivating the SIRT6 and AMPK/PGC-1α/AKT signaling pathways, reducing mitochondrial fission, and enhancing mitochondrial biogenesis and mitophagy [134]. Melatonin alleviates sepsis-induced cardiac dysfunction by increasing the SIRT3-mediated deacetylation of Beclin1 and promoting autophagy [135]. Melatonin has also been shown to attenuate H/R injury in H9c2 cells by inhibiting excessive activation of mitochondrial autophagy through the melatonin membrane receptor 2/SIRT3/FOXO3a signaling pathway [56]. Therefore, melatonin is anticipated to serve as a viable approach for the treatment of various cardiovascular diseases in the future (Table 1, Figure 4).

### 4.3. Antidiabetic and Lipid-Lowering Drugs

Multiple studies have shown that antidiabetic and lipid-lowering drugs exert notable cardioprotective effects. For instance, liraglutide activates SIRT1 to promote mitochondrial autophagy and reduce cellular oxidative stress, thereby maintaining mitochondrial homeostasis. Blocking the glucagon-like peptide-1 receptor or decreasing Parkin expression abolishes the beneficial effects on mitochondria [136]. Sitagliptin activates SIRT3 and suppresses excessive activation of autophagy induced by H/R to alleviate oxidative stress and mitochondrial dysfunction, thereby improving myocardial injury [137]. Metformin upregulates SIRT1 and AMPK to induce autophagy and thus improves severe complications of diabetes, including cardiac remodeling and heart failure [160]. Empagliflozin protects cardiomyocytes against DOX-induced cardiac toxicity by activating the Beclin1-TLR9-SIRT3 complex-mediated mitochondrial autophagy pathway [63]. Fenofibrate (FF), a peroxisome proliferator-activated receptor-α agonist, is employed for the clinical treatment of hypertriglyceridemia. FF prevents diabetes-induced cardiac dysfunction, inflammation, and cardiac remodeling and increases the expression of FGF21 and SIRT1 in both patients with and without diabetes. The knockout of FGF21, the inhibition of autophagy by 3-methyladenine (3MA), or the inhibition of SIRT1 by sirtinol abolishes the therapeutic effects of FF. These findings suggest that FF prevents type 1 diabetes-induced cardiac pathology and functional abnormalities by increasing FGF21 expression and upregulating SIRT1-mediated autophagy [138] (Table 1, Figure 4).

### 4.4. Phytochemicals

Studies have shown that certain phytochemicals exert a protective effect on car-diovascular diseases by modulating Sirtuin-mediated autophagy. For instance, quercetin prevents oxidative stress damage induced by H/R by regulating mitochondrial autophagy and ER stress through the SIRT1/TMBIM6 pathway [36]. Puerarin promotes autophagy, enhances mitochondrial antioxidant capacity, prevents excessive ROS production, suppresses the expression of inflammatory factors and oxidative stress damage, improves mitochondrial respiration and energy metabolism, and increases the susceptibility of HUVECs to an inflammatory state by increasing SIRT1 expression [139]. Gypenoside, salidroside, delphinidin-3-glucoside, paeoniflorin, araloside C, and ginsenoside Rb1 attenuate the progression of AS by activating the SIRT1-mediated autophagy pathway, inhibiting the uptake of ox-LDL and foam cell formation, regulating macrophage polarization, and alleviating endothelial damage [78,140,141,143,144,145]. Spinacetin and berberine enhance myocardial cell survival by regulating SIRT3-mediated autophagy, reducing myocardial enzyme and malondialdehyde levels, and protecting against DOX-induced cardiac toxicity in rats. The latter exerts a dual effect on mitochondrial autophagy, contingent upon the concentration of DOX [53,142]. Berberine promotes autophagy in peritoneal macrophages by activating the nicotinamide adenine dinucleotide (NAD^+^)/SIRT1/TFEB pathway and inhibiting the PI3K/AKT/mTOR signaling pathway, thereby suppressing macrophage apoptosis [34]. In a mouse model of myocardial infarction, Tongguan capsule has been demonstrated to promote autophagy by increasing the expression of SIRT1, reducing the phosphorylation of mTOR and its downstream effectors, namely 70 kDa ribosomal protein S6 kinase and 4E binding protein 1, and preventing the inflammation and apoptosis of myocardial cells, thereby improving cardiac remodeling [148]. Daming capsule has been shown to upregulate the expression of the mitochondrial autophagy receptor nucleotide-binding oligomerization domain-like receptor family member X1 by activating the SIRT1/AMPK signaling pathway, increase mitochondrial autophagy, and inhibit oxidative stress and inflammatory responses in myocardial cells, thereby improving heart function following myocardial infarction [149]. Additionally, ginsenoside Rb2 exhibits a protective effect against MI/R injury by inducing SIRT1 expression, reducing myocardial inflammation, and alleviating oxidative stress [146]. In both in vitro and in vivo models of cardiac aging, acacetin has been shown to reduce the production of advanced glycation end products, shorten myocardial telomere length, and decrease the expression of cellular senescence markers and mitochondrial autophagy signaling proteins in a dose- and concentration-dependent manner, thereby improving cardiac function. Additional research has demonstrated that acacetin activates the SIRT1-mediated SIRT6/AMPK signaling pathway to enhance mitochondrial autophagy, maintain mitochondrial function, and inhibit cardiac aging [150]. Recent studies have revealed that ginsenoside Rg1 enhances SIRT1-mediated mitochondrial autophagy to alleviate cardiac remodeling, reduce mitochondrial damage, and improve cardiac function [147]. Ginkgolide B enhances autophagy via the SIRT1/FOXO1 signaling pathway, inhibits Ang II-induced myocardial hypertrophy, and may potentially serve as a therapeutic modality for pathological cardiac hypertrophy [151]. In addition, ginkgolide B has been demonstrated to protect isolated hearts against arrhythmias induced by ischemia but not reperfusion, which appears to be associated with an antagonism antagonistic increase in slow calcium influx in the myocardium. However, it is unclear whether Sirtuin-mediated autophagy is involved in anti-arrhythmias induced by ischaemic [90] (Table 1, Figure 4).

### 4.5. Novel Small Molecules

ZLN005, a novel small molecule, restores autophagy in cardiomyocytes inhibited by high glucose levels, enhances cell viability, and alleviates oxidative damage by enhancing the SIRT3-mediated pathway. However, the protective effect of ZLN005 in cardiomyocytes treated with high concentrations of glucose is attenuated by the SIRT3 inhibitor EX-527. These findings suggest that ZLN005 inhibits cardiomyocyte damage induced by high glucose levels by upregulating SIRT1 expression and autophagy [152]. MLN4924, an inhibitor of neddylation, alleviates left ventricular systolic dysfunction, limits myocardial infarct size in mice with MIRI, and restores defective autophagic flux in H_2_O_2_-treated cells. Further investigations have shown that MLN4924 restores impaired autophagic flux by regulating SIRT1 expression [153] (Table 1, Figure 4).

### 4.6. Gas Molecules

H_2_S is a novel member of the gasotransmitter family, exhibiting functional similarities to NO and CO. H_2_S delays the onset of AS by safeguarding endothelial cells through SIRT1/FOXO1-mediated autophagy [155]. In addition, research has shown that H2 alleviates ox-LDL-induced inflammation by stimulating autophagy through SIRT1 [156]. Sevoflurane increases SIRT1 levels during MI/R, promotes LC3 deacetylation, and enhances autophagy, thereby improving myocardial cell damage [154] (Table 1, Figure 4).

### 4.7. Physical Approaches

Electrical stimulation (ES) is a non-invasive and safe therapeutic modality that reduces inflammatory cytokine release and ROS production by downregulating the NLRP3 inflammasome, reversing SIRT3 downregulation, deacetylating ATG5, and inducing autophagy. Additional investigations have revealed that ES promotes autophagy by increasing SIRT3 expression and inhibiting ROS production and inflammatory cytokine release, thereby counteracting AS [157]. Additionally, studies have demonstrated that ES reduces lipid accumulation, inhibits the secretion of inflammatory factors, and restores proper macrophage autophagy through SIRT1/ATG5 pathway-mediated autophagy, thereby reducing lesion formation in AS [158] (Table 1, Figure 4).

### 4.8. Other Therapeutic Drugs

Feeding spontaneously hypertensive rats with α-linolenic acid lowers blood pressure, increases SIRT3 expression, reduces MnSOD2 acetylation, restores autophagy, and inhibits vascular oxidative stress, thereby improving endothelial dysfunction and reducing blood pressure [16]. Ursolic acid protects HUVECs from ox-LDL-induced cytotoxicity and exerts anti-AS effects by increasing SIRT1 expression in the cells, decreasing the acetylation of lysine residues in ATG5, and enhancing autophagy [159]. NAD^+^, a regulator and target of autophagy, plays a crucial role in coordinating the cell stress response [161]. Nicotinamide activates the NAD^+^/SIRT1 signaling pathway, restores autophagic flux in myocardial cells, enhances lysosomal clearance, and alleviates oxidative stress, thereby preventing DOX-induced cardiac toxicity [113]. In addition, recent studies have demonstrated that SQ 26533, nicainoprol, cannabidiol, gluconolactone, endothelin-1, and caspase inhibitors can alleviate MI/R damage and counteract subsequent arrhythmia; however, determining whether Sirtuin-mediated autophagy is involved in the cardioprotective effect of the novel drugs mentioned requires further investigation [162,163,164,165,166,167,168] (Table 1, Figure 4).

## 5. Conclusions

Sirtuin-mediated autophagy is a promising therapeutic target for cardiovascular diseases. Upon stimulation by endogenous and exogenous factors, Sirtuins regulate multiple signaling pathways at the levels of transcription, post-transcription, and post-translational modification to eliminate damaged cells and organelles, maintain metabolic homeostasis, suppress inflammation, counteract apoptosis, inhibit oxidative stress, promote myocardial regeneration, and repair, and revert myocardial hypertrophy, thus playing a protective role in various cardiovascular diseases. However, autophagy is a highly dynamic process that is regulated by diverse internal and external environmental factors. Moreover, the characteristics of the different stages of autophagy require a range of techniques and methodologies for accurate measurement. Consequently, in vivo quantification of autophagy remains challenging. Researchers must integrate multiple experimental methods, establish multiple disease models, and leverage multiple clinical samples to gain pivotal insights into the role of Sirtuin-mediated autophagy in cardiovascular diseases. Presently, the functional relationship and the precise mechanism underpinning Sirtuins and cardiovascular autophagy are yet to be fully understood. The continuing exploration of the interaction between Sirtuins and autophagy, coupled with the pursuit of developing novel Sirtuins modulators, holds potential for the discovery and development of effective anti-cardiovascular drugs.

## Figures and Tables

**Table 1:** Advances in Sirtuins/autophagy-based therapies.

Treatment	Mechanism	Cardiovascular Diseases	References
Resveratrol	SIRT1/autophagy SIRT3/autophagy SIRT1/FOXO1/autophagy cAMP/PRKA/AMPK/SIRT1/autophagy	Heart failure; AS; artery thrombosis; MI/R; DCM; MTHFR deficiency	[32,82,125,126,127,128,129,130,131]
Melatonin	SIRT1/autophagy SIRT3/FOXO3a/autophagy SIRT3/NRF2 SIRT6/AMPK-PGC-1α-AKT	Heart failure; AS; DCM; MI/R; myocardial damage; hypertension;	[15,56,83,132,133,134,135]
Liraglutide	SIRT1/autophagy	MI	[136]
Empagliflozin	Beclin1-TLR9-SIRT3 complexes	Drug-induced cardiac injury	[63]
Sitagliptin	SIRT3/autophagy	H/R	[137]
Fenofibrate	FGF21/SIRT1/autophagy	DCM	[138]
Quercetin	SIRT1/TMBIM6	H/R	[36]
Puerarin	SIRT1/autophagy	AS	[139]
Gypenoside	SIRT1/FOXO1/autophagy	AS	[140]
Delphinidin-3-glucoside	AMPK/SIRT1/autophagy	AS	[141]
Berberine	NAD^+^/SIRT1/TFEB/autophagy PI3K/AKT/mTOR/autophagy SIRT3/autophagy	AS; drug-induced cardiac injury	[34,142]
Araloside C	SIRT1/autophagy	AS	[78]
Salidroside	SIRT1/FOXO1/autophagy	AS	[143]
Paeoniflorin	SIRT1/autophagy	AS	[144]
Ginsenoside Rb1	SIRT1/Beclin1/autophagy	AS	[145]
Ginsenoside Rb2	SIRT1/autophagy	MI/R	[146]
Ginsenoside Rg1	SIRT1/PINK1/Parkin/mitophagy	Heart failure	[147]
Spinacetin	SIRT3/AMPK/mTOR autophagy	Drug-induced cardiac injury	[53]
Tongguan Capsule	SIRT1/mTOR/P70S6K/4EBP1/autophagy	MI	[148]
Daming Capsule	SIRT1/AMPK/NLRX1/autophagy	MI	[149]
Acacetin	SIRT1-SIRT6/AMPK/autophagy	Cardiac senescence	[150]
Ginkgolide B	SIRT1/FOXO1/autophagy	Heart failure	[151]
ZLN005	SIRT3/autophagy	DCM	[152]
MLN4924	SIRT1/autophagy	MI/R	[153]
Sevoflurane	SIRT1/autophagy	MI/R	[154]
H2S and H2	SIRT1/autophagy	AS	[155,156]
Electrical stimulation	SIRT1/Atg5 SIRT3/autophagy	AS	[157,158]
α-Linolenic acid	SIRT3/autophagy	Hypertension	[16]
Ursolic acid	SIRT1/autophagy	AS	[159]
Nicotinamide	NAD^+^/SIRT1/autophagy	Drug-induced Cardiac Injury	[113]

Table 1 Medical and physical therapies based Sirtuin-mediated autophagy in diverse cardiovascular diseases. AKT, protein kinase B; AMPK, adeno sine 5′-monophosphate (AMP)-activated protein kinase; AS, atherosclerosis; ATG5, autophagy-related 5; cAMP, 3′–5′-cyclic adenosine monophosphate; DCM, diabetic cardiomyopathy; FGF21, fibroblast growth factor 21; FOXO1, the forkhead box-1; FOXO3a, the forkhead box O3a; H/R, hypoxia/reoxygenation; mTOR, mammalian target of rapamycin; MI, myocardial infarction; MI/R, myocardial ischemia-reperfusion; NAD^+^, nicotinamide adenine dinucleotide; PARK2, parkin RBR E3 ubiquitin protein ligase; P70S6K, 70 kDa ribosomal protein S6 kinase; NLRX1, nucleotide-binding oligomerization domain-like receptor family member X1; NRF2, the nuclear factor E2-related factor 2; PI3K, phosphoinositide 3-kinase; 4EBP1, 4E binding protein 1; TFEB, transcription factor EB; TLR9, the Toll-like receptor 9; TMBIM6, transmembrane BAX inhibitor motif containing 6.

## Data Availability

Not applicable.

## Abbreviations

The following abbreviations are used in this manuscript:

AR	androgen receptor
ATG5	autophagy-related 5
ATG7	autophagy-related 7
ATG12	autophagy-related 12
BNIP3	BCL2 interacting protein 3
CPS1	carbamoyl-phosphate synthase 1
EX-527	SIRT1 inhibitor
JNK	c-Jun amino-terminal kinase
Nkx3.2	NK3 homeobox 2
PARK2	parkin RBR E3 ubiquitin protein ligase
PCSK6	proprotein convertase subtilisin/kexin type 6
PINK	PTEN induced putative kinase
17β-E2	17beta-estradiol
3MA	3-methyladenine;
4EBP1	4E binding protein 1
AK-7	SIRT2 inhibitor;
AKT	protein kinase B
AMPK	adenosine 5′-monophosphate (AMP)-activated protein kinase
Ang II	angiotensin II
AS	atherosclerosis
ATG12	autophagy related 12
ATG5	autophagy related 5
Bnip	BCL2 interacting protein
cAMP	3′-5′-cyclic adenosine monophosphate
CHMP2B	CHarged Multivesicular body Protein 2B
circRNA	circular RNA
CREB	cAMP-response element binding protein
DCM	diabetic cardiomyopathy;
DOX	doxorubicin;
eEF2	eukaryotic elongation factor 2
eEF2k	eukaryotic elongation factor 2 kinase
EndoMT	endothelial-to-mesenchymal transition
EPC	endothelial progenitor cell
ERK	extracellular regulated kinase
ERS	endoplasmic reticulum stress
EX-527	SIRT1 inhibitor
FAT10	human leukocyte antigen-F adjacent transcript 10
FF	fenofibrate
FGF21	fibroblast growth factor 21
FOXM1	the forkhead box M1
FOXO1	the forkhead box-1
FOXO3a	the forkhead box O3a
GATA5	GATA-binding protein 5
GLS	glutaminase
GTSP1	glutathione S-transferase P1
H/R	hypoxia–reoxygenation
Hes-1	hairy and Enhancer of split homolog-1
HUVECs	human umbilical vein endothelial cells
IGF2	insulin-like growth factor 2
IL-1B	interleukin-1B
LC3	microtubule-associated protein light chain 3
LDHB	lactate dehydrogenase B
LKB1	liver kinase B1
LncRNA PVT1	Long noncoding RNA PVT1
LncRNA	long noncoding RNA
MDA	malondialdehyde
MDL800	SIRT6 activator
MFN2	mitofusin 2
MI	myocardial infarction
MI/R	myocardial ischemia/reperfusion
MnSOD2	manganese superoxide dismutase 2
Mst1	macrophage stimulating 1
MT2	melatonin membrane receptor 2
MTHFR	methylenetetrahydrofolate-reductase
mTOR	mammalian target of rapamycin
NAD^+^	nicotinamide adenine dinucleotide
Nampt	nicotinamide phosphoribosyl transferase
NLRP3	NOD-like receptor family pyrin domain containing 3
NLRX1	nucleotide-binding oligomerization domain-like receptor family member X1
NRF2	the nuclear factor E2-related factor 2
OPA1	optic atrophy 1
ox-LDL	oxidative modification of low-density lipoprotein
P70S6K	70 kDa ribosomal protein S6 kinase
PARK2	parkin RBR E3 ubiquitin protein ligase
PGC-1α	peroxisome proliferator-activated receptor γ coactivator 1-α
PI3K	phosphoinositide 3-kinase
PICK1	protein interacting with C kinase 1
PINK	PTEN induced putative kinase
ROS	reactive oxygen species
RSV	resveratrol
SCFD1	sec1 family domain containing 1
SIRTs	silent information regulators
SMAD4	mothers against decapentaplegic homolog 4
SQSTM1	sequestosome
STAT3	signal transducer and activator of transcription 3
SUMO1	small ubiquitin-like modifier 1
TFEB	transcription factor EB
TGFBR1	type1 transforming growth factor beta receptor
TLR9	the Toll-like receptor 9
TMBIM6	transmembrane Bax inhibitor Motif Containing 6
TNF-α	tumor necrosing factor alpha
ULK1	unc-51-like autophagy activating kinase 1
vWf	von Willebrand facto
YAP	yes-associated protein

## References

[1] Rine J., Herskowitz I. (1987). Four genes responsible for a position effect on expression from HML and HMR in Saccharomyces cerevisiae. Genetics.

[2] Li P., Ge J., Li H. (2020). Lysine acetyltransferases and lysine deacetylases as targets for cardiovascular disease. Nat. Rev. Cardiol..

[3] Wu Q.J., Zhang T.N., Chen H.H., Yu X.F., Lv J.L., Liu Y.Y., Liu Y.S., Zheng G., Zhao J.Q., Wei Y.F. (2022). The sirtuin family in health and disease. Signal Transduct. Target. Ther..

[4] Aventaggiato M., Vernucci E., Barreca F., Russo M.A., Tafani M. (2021). Sirtuins’ control of autophagy and mitophagy in cancer. Pharmacol. Ther..

[5] Sciarretta S., Hariharan N., Monden Y., Zablocki D., Sadoshima J. (2011). Is autophagy in response to ischemia and reperfusion protective or detrimental for the heart?. Pediatr. Cardiol..

[6] Sharma V., Verma S., Seranova E., Sarkar S., Kumar D. (2018). Selective Autophagy and Xenophagy in Infection and Disease. Front. Cell Dev. Biol..

[7] Bravo-San Pedro J.M., Kroemer G., Galluzzi L. (2017). Autophagy and Mitophagy in Cardiovascular Disease. Circ. Res..

[8] Conti V., Forte M., Corbi G., Russomanno G., Formisano L., Landolfi A., Izzo V., Filippelli A., Vecchione C., Carrizzo A. (2017). Sirtuins: Possible Clinical Implications in Cardio and Cerebrovascular Diseases. Curr. Drug Targets.

[9] Baeken M.W. (2023). Sirtuins and their influence on autophagy. J. Cell. Biochem..

[10] Lee I.H. (2019). Mechanisms and disease implications of sirtuin-mediated autophagic regulation. Exp. Mol. Med..

[11] Packer M. (2020). Longevity genes, cardiac ageing, and the pathogenesis of cardiomyopathy: Implications for understanding the effects of current and future treatments for heart failure. Eur. Heart J..

[12] Grootaert M.O.J., Bennett M.R. (2022). Sirtuins in atherosclerosis: Guardians of healthspan and therapeutic targets. Nat. Rev. Cardiol..

[13] Zheng Y., Shi B., Ma M., Wu X., Lin X. (2019). The novel relationship between Sirt3 and autophagy in myocardial ischemia-reperfusion. J. Cell. Physiol..

[14] Li H., Zheng F., Zhang Y., Sun J., Gao F., Shi G. (2022). Resveratrol, novel application by preconditioning to attenuate myocardial ischemia/reperfusion injury in mice through regulate AMPK pathway and autophagy level. J. Cell. Mol. Med..

[15] Balarastaghi S., Barangi S., Hosseinzadeh H., Imenshahidi M., Moosavi Z., Razavi B.M., Karimi G. (2022). Melatonin improves arsenic-induced hypertension through the inactivation of the Sirt1/autophagy pathway in rat. Biomed. Pharmacother..

[16] Li G., Wang X., Yang H., Zhang P., Wu F., Li Y., Zhou Y., Zhang X., Ma H., Zhang W. (2020). α-Linolenic acid but not linolenic acid protects against hypertension: Critical role of SIRT3 and autophagic flux. Cell Death Dis..

[17] Qiu Z., Ming H., Zhang Y., Yu Y., Lei S., Xia Z.Y. (2022). The Protective Role of Bmal1-Regulated Autophagy Mediated by HDAC3/SIRT1 Pathway in Myocardial Ischemia/Reperfusion Injury of Diabetic Rats. Cardiovasc. Drugs Ther..

[18] Govender J., Loos B., Marais E., Engelbrecht A.M. (2014). Mitochondrial catastrophe during doxorubicin-induced cardiotoxicity: A review of the protective role of melatonin. J. Pineal Res..

[19] Jiang X., Zhang K., Gao C., Ma W., Liu M., Guo X., Bao G., Han B., Hu H., Zhao Z. (2022). Activation of FMS-like tyrosine kinase 3 protects against isoprenaline-induced cardiac hypertrophy by improving autophagy and mitochondrial dynamics. FASEB J..

[20] Chen D., Zheng K., Wu H., Zhang X., Ye W., Tan X., Xiong Y. (2021). Lin28a attenuates cerebral ischemia/reperfusion injury through regulating Sirt3-induced autophagy. Brain Res. Bull..

[21] Hao Y., Lu Q., Yang G., Ma A. (2016). Lin28a protects against postinfarction myocardial remodeling and dysfunction through Sirt1 activation and autophagy enhancement. Biochem. Biophys. Res. Commun..

[22] Hariharan N., Maejima Y., Nakae J., Paik J., Depinho R.A., Sadoshima J. (2010). Deacetylation of FoxO by Sirt1 Plays an Essential Role in Mediating Starvation-Induced Autophagy in Cardiac Myocytes. Circ. Res..

[23] Sasaki Y., Ikeda Y., Uchikado Y., Akasaki Y., Sadoshima J., Ohishi M. (2021). Estrogen Plays a Crucial Role in Rab9-Dependent Mitochondrial Autophagy, Delaying Arterial Senescence. J. Am. Heart Assoc..

[24] Liu Q., Li H., Wang J., Zhong L., Chen X., Zhang R., Wang H. (2020). Glucose restriction delays senescence and promotes proliferation of HUVECs via the AMPK/SIRT1-FOXA3-Beclin1 pathway. Exp. Gerontol..

[25] Li C., Guo Z., Liu F., An P., Wang M., Yang D., Tang Q. (2023). PCSK6 attenuates cardiac dysfunction in doxorubicin-induced cardiotoxicity by regulating autophagy. Free Radic. Biol. Med..

[26] Ning S., Li Z., Ji Z., Fan D., Wang K., Wang Q., Hua L., Zhang J., Meng X., Yuan Y. (2020). MicroRNA-494 suppresses hypoxia/reoxygenation-induced cardiomyocyte apoptosis and autophagy via the PI3K/AKT/mTOR signaling pathway by targeting SIRT1. Mol. Med. Rep..

[27] Zhan H., Huang F., Niu Q., Jiao M., Han X., Zhang K., Ma W., Mi S., Guo S., Zhao Z. (2021). Downregulation of miR-128 Ameliorates Ang II-Induced Cardiac Remodeling via SIRT1/PIK3R1 Multiple Targets. Oxid. Med. Cell. Longev..

[28] Qi Y., Zhang K., Li P., Wu Z. (2021). Down-regulating miR-217-5p Protects Cardiomyocytes against Ischemia/Reperfusion Injury by Restoring Mitochondrial Function via Targeting SIRT1. Inflammation.

[29] Guo Y., Yang J.H., Cao S.D., Gao C.X., He Y., Wang Y., Wan H.T., Jin B. (2021). Effect of main ingredients of Danhong Injection against oxidative stress induced autophagy injury via miR-19a/SIRT1 pathway in endothelial cells. Phytomedicine.

[30] Wang W., Wang L., Yang M., Wu C., Lan R., Wang W., Li Y. (2021). Circ-SIRT1 inhibits cardiac hypertrophy via activating SIRT1 to promote autophagy. Cell Death Dis..

[31] Yang J., Lin X., Wang L., Sun T., Zhao Q., Ma Q., Zhou Y. (2020). LncRNA MALAT1 Enhances ox-LDL-Induced Autophagy through the SIRT1/MAPK/NF-κB Pathway in Macrophages. Curr. Vasc. Pharmacol..

[32] Wang B., Yang Q., Sun Y.Y., Xing Y.F., Wang Y.B., Lu X.T., Bai W.W., Liu X.Q., Zhao Y.X. (2014). Resveratrol-enhanced autophagic flux ameliorates myocardial oxidative stress injury in diabetic mice. J. Cell. Mol. Med..

[33] Packer M. (2020). Cardioprotective Effects of Sirtuin-1 and Its Downstream Effectors: Potential Role in Mediating the Heart Failure Benefits of SGLT2 (Sodium-Glucose Cotransporter 2) Inhibitors. Circ. Heart Fail..

[34] Zheng Y., Kou J., Wang P., Ye T., Wang Z., Gao Z., Cong L., Li M., Dong B., Yang W. (2021). Berberine-induced TFEB deacetylation by SIRT1 promotes autophagy in peritoneal macrophages. Aging.

[35] Pires Da Silva J., Monceaux K., Guilbert A., Gressette M., Piquereau J., Novotova M., Ventura-Clapier R., Garnier A., Lemaire C. (2020). SIRT1 Protects the Heart from ER Stress-Induced Injury by Promoting eEF2K/eEF2-Dependent Autophagy. Cells.

[36] Chang X., Zhang T., Meng Q., Wang S., Yan P., Wang X., Luo D., Zhou X., Ji R. (2021). Quercetin Improves Cardiomyocyte Vulnerability to Hypoxia by Regulating SIRT1/TMBIM6-Related Mitophagy and Endoplasmic Reticulum Stress. Oxid. Med. Cell. Longev..

[37] Wan R., Yuan P., Guo L., Shao J., Liu X., Lai W., Kong Q., Chen L., Ge J., Xu Z. (2021). Ubiquitin-like protein FAT10 suppresses SIRT1-mediated autophagy to protect against ischemic myocardial injury. J. Mol. Cell. Cardiol..

[38] Yuan P., Hu Q., He X., Long Y., Song X., Wu F., He Y., Zhou X. (2020). Laminar flow inhibits the Hippo/YAP pathway via autophagy and SIRT1-mediated deacetylation against atherosclerosis. Cell Death Dis..

[39] Takeda-Watanabe A., Kitada M., Kanasaki K., Koya D. (2012). SIRT1 inactivation induces inflammation through the dysregulation of autophagy in human THP-1 cells. Biochem. Biophys. Res. Commun..

[40] Sundaresan N.R., Vasudevan P., Zhong L., Kim G., Samant S., Parekh V., Pillai V.B., Ravindra P.V., Gupta M., Jeevanandam V. (2012). The sirtuin SIRT6 blocks IGF-Akt signaling and development of cardiac hypertrophy by targeting c-Jun. Nat. Med..

[41] Lu J., Sun D., Liu Z., Li M., Hong H., Liu C., Gao S., Li H., Cai Y., Chen S. (2016). SIRT6 suppresses isoproterenol-induced cardiac hypertrophy through activation of autophagy. Transl. Res..

[42] Song J.J., Yang M., Liu Y., Song J.W., Wang J., Chi H.J., Liu X.Y., Zuo K., Yang X.C., Zhong J.C. (2020). MicroRNA-122 aggravates angiotensin II-mediated apoptosis and autophagy imbalance in rat aortic adventitial fibroblasts via the modulation of SIRT6-elabela-ACE2 signaling. Eur. J. Pharmacol..

[43] Zhao Y., Jia X., Yang X., Bai X., Lu Y., Zhu L., Cheng W., Shu M., Zhu Y., Du X. (2022). Deacetylation of Caveolin-1 by Sirt6 induces autophagy and retards high glucose-stimulated LDL transcytosis and atherosclerosis formation. Metabolism.

[44] Li X., Liu L., Jiang W., Liu M., Wang Y., Ma H., Mu N., Wang H. (2022). SIRT6 Protects Against Myocardial Ischemia-Reperfusion Injury by Attenuating Aging-Related CHMP2B Accumulation. J. Cardiovasc. Transl. Res..

[45] Guo J., Wang Z., Wu J., Liu M., Li M., Sun Y., Huang W., Li Y., Zhang Y., Tang W. (2019). Endothelial SIRT6 Is Vital to Prevent Hypertension and Associated Cardiorenal Injury Through Targeting Nkx3.2-GATA5 Signaling. Circ. Res..

[46] Ford E., Voit R., Liszt G., Magin C., Grummt I., Guarente L. (2006). Mammalian Sir2 homolog SIRT7 is an activator of RNA polymerase I transcription. Genes Dev..

[47] Michishita E., Park J.Y., Burneskis J.M., Barrett J.C., Horikawa I. (2005). Evolutionarily conserved and nonconserved cellular localizations and functions of human SIRT proteins. Mol. Biol. Cell.

[48] Yu W., Cui X., Wan Z., Yu Y., Liu X., Jin L. (2018). Silencing forkhead box M1 promotes apoptosis and autophagy through SIRT7/mTOR/IGF2 pathway in gastric cancer cells. J. Cell. Biochem..

[49] Wu S.Y., Du Y.C., Yue C.F. (2020). Sirt7 protects chondrocytes degeneration in osteoarthritis via autophagy activation. Eur. Rev. Med. Pharmacol. Sci..

[50] Ding M., Jiang C.Y., Zhang Y., Zhao J., Han B.M., Xia S.J. (2020). SIRT7 depletion inhibits cell proliferation and androgen-induced autophagy by suppressing the AR signaling in prostate cancer. J. Exp. Clin. Cancer Res..

[51] Araki S., Izumiya Y., Rokutanda T., Ianni A., Hanatani S., Kimura Y., Onoue Y., Senokuchi T., Yoshizawa T., Yasuda O. (2015). Sirt7 Contributes to Myocardial Tissue Repair by Maintaining Transforming Growth Factor-β Signaling Pathway. Circulation.

[52] Hu J., Liu T., Fu F., Cui Z., Lai Q., Zhang Y., Yu B., Liu F., Kou J., Li F. (2022). Omentin1 ameliorates myocardial ischemia-induced heart failure via SIRT3/FOXO3a-dependent mitochondrial dynamical homeostasis and mitophagy. J. Transl. Med..

[53] Liu D., Zhao L. (2022). Spinacetin alleviates doxorubicin-induced cardiotoxicity by initiating protective autophagy through SIRT3/AMPK/mTOR pathways. Phytomedicine.

[54] Fan X., He Y., Wu G., Chen H., Cheng X., Zhan Y., An C., Chen T., Wang X. (2023). Sirt3 activates autophagy to prevent DOX-induced senescence by inactivating PI3K/AKT/mTOR pathway in A549 cells. Biochim. Biophys. Acta. Mol. Cell Res..

[55] Ma C., Zhao Y., Ding X., Gao B. (2022). The role of Sirt3 in the changes of skeletal muscle mitophagy induced by hypoxic training. Gen. Physiol. Biophys..

[56] Wu J., Yang Y., Gao Y., Wang Z., Ma J. (2020). Melatonin Attenuates Anoxia/Reoxygenation Injury by Inhibiting Excessive Mitophagy Through the MT2/SIRT3/FoxO3a Signaling Pathway in H9c2 Cells. Drug Des. Dev. Ther..

[57] Yang Y., Li N., Chen T., Zhang C., Li J., Liu L., Qi Y., Zheng X., Zhang C., Bu P. (2019). Sirt3 promotes sensitivity to sunitinib-induced cardiotoxicity via inhibition of GTSP1/JNK/autophagy pathway in vivo and in vitro. Arch. Toxicol..

[58] Wang Y., Chang J., Wang Z.Q., Li Y. (2021). Sirt3 promotes the autophagy of HK-2 human proximal tubular epithelial cells via the inhibition of Notch-1/Hes-1 signaling. Mol. Med. Rep..

[59] Li R., Xin T., Li D., Wang C., Zhu H., Zhou H. (2018). Therapeutic effect of Sirtuin 3 on ameliorating nonalcoholic fatty liver disease: The role of the ERK-CREB pathway and Bnip3-mediated mitophagy. Redox Biol..

[60] Zhang M., Lin J., Wang S., Cheng Z., Hu J., Wang T., Man W., Yin T., Guo W., Gao E. (2017). Melatonin protects against diabetic cardiomyopathy through Mst1/Sirt3 signaling. J. Pineal Res..

[61] Xiang X., Wang Y., Huang G., Huang J., Gao M., Sun M., Xia H., Pare R., Li J., Ruan Y. (2023). 17β-estradiol suppresses H_2_O_2_-induced senescence in human umbilical vein endothelial cells by inducing autophagy through the PVT1/miR-31/SIRT3 axis. J. Steroid Biochem. Mol. Biol..

[62] Xiang X., Huang J., Song S., Wang Y., Zeng Y., Wu S., Ruan Y. (2020). 17β-estradiol inhibits H_2_O_2_-induced senescence in HUVEC cells through upregulating SIRT3 expression and promoting autophagy. Biogerontology.

[63] Wang C.Y., Chen C.C., Lin M.H., Su H.T., Ho M.Y., Yeh J.K., Tsai M.L., Hsieh I.C., Wen M.S. (2020). TLR9 Binding to Beclin 1 and Mitochondrial SIRT3 by a Sodium-Glucose Co-Transporter 2 Inhibitor Protects the Heart from Doxorubicin Toxicity. Biology.

[64] Zhang L., Ma C., Wang X., Bai J., He S., Zhang J., Xin W., Li Y., Jiang Y., Li J. (2020). MicroRNA-874-5p regulates autophagy and proliferation in pulmonary artery smooth muscle cells by targeting Sirtuin 3. Eur. J. Pharmacol..

[65] Lang A., Anand R., Altinoluk-Hambüchen S., Ezzahoini H., Stefanski A., Iram A., Bergmann L., Urbach J., Böhler P., Hänsel J. (2017). SIRT4 interacts with OPA1 and regulates mitochondrial quality control and mitophagy. Aging.

[66] He L., Wang J., Yang Y., Zou P., Xia Z., Li J. (2022). SIRT4 Suppresses Doxorubicin-Induced Cardiotoxicity by Regulating the AKT/mTOR/Autophagy Pathway. Toxicology.

[67] Huang H., Ouyang Q., Mei K., Liu T., Sun Q., Liu W., Liu R. (2023). Acetylation of SCFD1 regulates SNARE complex formation and autophagosome-lysosome fusion. Autophagy.

[68] Nakagawa T., Lomb D.J., Haigis M.C., Guarente L. (2009). SIRT5 Deacetylates carbamoyl phosphate synthetase 1 and regulates the urea cycle. Cell.

[69] Polletta L., Vernucci E., Carnevale I., Arcangeli T., Rotili D., Palmerio S., Steegborn C., Nowak T., Schutkowski M., Pellegrini L. (2015). SIRT5 regulation of ammonia-induced autophagy and mitophagy. Autophagy.

[70] Shi L., Yan H., An S., Shen M., Jia W., Zhang R., Zhao L., Huang G., Liu J. (2019). SIRT5-mediated deacetylation of LDHB promotes autophagy and tumorigenesis in colorectal cancer. Mol. Oncol..

[71] Ng F., Tang B.L. (2013). Sirtuins’ modulation of autophagy. J. Cell. Physiol..

[72] Wang L., Xu P., Xie X., Hu F., Jiang L., Hu R., Ding F., Xiao H., Zhang H. (2020). Down Regulation of SIRT2 Reduced ASS Induced NSCLC Apoptosis Through the Release of Autophagy Components via Exosomes. Front. Cell Dev. Biol..

[73] Tang X., Chen X.F., Wang N.Y., Wang X.M., Liang S.T., Zheng W., Lu Y.B., Zhao X., Hao D.L., Zhang Z.Q. (2017). SIRT2 Acts as a Cardioprotective Deacetylase in Pathological Cardiac Hypertrophy. Circulation.

[74] Lynn E.G., McLeod C.J., Gordon J.P., Bao J., Sack M.N. (2008). SIRT2 is a negative regulator of anoxia-reoxygenation tolerance via regulation of 14-3-3 zeta and BAD in H9c2 cells. FEBS Lett..

[75] Roychowdhury S., Gandhirajan A., Kibler C., Wang X., Vachharajani V. (2021). Sirtuin 2 Dysregulates Autophagy in High-Fat-Exposed Immune-Tolerant Macrophages. Cells.

[76] Liu G., Park S.H., Imbesi M., Nathan W.J., Zou X., Zhu Y., Jiang H., Parisiadou L., Gius D. (2017). Loss of NAD-Dependent Protein Deacetylase Sirtuin-2 Alters Mitochondrial Protein Acetylation and Dysregulates Mitophagy. Antioxid. Redox Signal..

[77] Kitada M., Ogura Y., Koya D. (2016). The protective role of Sirt1 in vascular tissue: Its relationship to vascular aging and atherosclerosis. Aging.

[78] Luo Y., Lu S., Gao Y., Yang K., Wu D., Xu X., Sun G., Sun X. (2020). Araloside C attenuates atherosclerosis by modulating macrophage polarization via Sirt1-mediated autophagy. Aging.

[79] Hassanpour M., Rezabakhsh A., Pezeshkian M., Rahbarghazi R., Nouri M. (2018). Distinct role of autophagy on angiogenesis: Highlights on the effect of autophagy in endothelial lineage and progenitor cells. Stem Cell Res. Ther..

[80] Wang C., Mao C., Lou Y., Xu J., Wang Q., Zhang Z., Tang Q., Zhang X., Xu H., Feng Y. (2018). Monotropein promotes angiogenesis and inhibits oxidative stress-induced autophagy in endothelial progenitor cells to accelerate wound healing. J. Cell. Mol. Med..

[81] Li Y., Cui W., Song B., Ye X., Li Z., Lu C. (2022). Autophagy-Sirtuin1(SIRT1) Alleviated the Coronary Atherosclerosis (AS)in Mice through Regulating the Proliferation and Migration of Endothelial Progenitor Cells (EPCs) via wnt/β-catenin/GSK3β Signaling Pathway. J. Nutr. Health Aging.

[82] Wu Q., Hu Y., Jiang M., Wang F., Gong G. (2019). Effect of Autophagy Regulated by Sirt1/FoxO1 Pathway on the Release of Factors Promoting Thrombosis from Vascular Endothelial Cells. Int. J. Mol. Sci..

[83] Ma S., Chen J., Feng J., Zhang R., Fan M., Han D., Li X., Li C., Ren J., Wang Y. (2018). Melatonin Ameliorates the Progression of Atherosclerosis via Mitophagy Activation and NLRP3 Inflammasome Inhibition. Oxid. Med. Cell. Longev..

[84] He J., Zhang G., Pang Q., Yu C., Xiong J., Zhu J., Chen F. (2017). SIRT6 reduces macrophage foam cell formation by inducing autophagy and cholesterol efflux under ox-LDL condition. FEBS J..

[85] Wang T., Sun C., Hu L., Gao E., Li C., Wang H., Sun D. (2020). Sirt6 stabilizes atherosclerosis plaques by promoting macrophage autophagy and reducing contact with endothelial cells. Biochem. Cell Biol..

[86] Zi Y., Yi-An Y., Bing J., Yan L., Jing T., Chun-Yu G., Fan P., Hao L., Jia-Ni T., Han-Jin H. (2019). Sirt6-induced autophagy restricted TREM-1-mediated pyroptosis in ox-LDL-treated endothelial cells: Relevance to prognostication of patients with acute myocardial infarction. Cell Death Discov..

[87] Su G., Yang W., Wang S., Geng C., Guan X. (2021). SIRT1-autophagy axis inhibits excess iron-induced ferroptosis of foam cells and subsequently increases IL-1Β and IL-18. Biochem. Biophys. Res. Commun..

[88] Garlick P.B., Davies M.J., Hearse D.J., Slater T.F. (1987). Direct detection of free radicals in the reperfused rat heart using electron spin resonance spectroscopy. Circ. Res..

[89] Liu Y., Zhang J., Zhang D., Yu P., Zhang J., Yu S. (2022). Research Progress on the Role of Pyroptosis in Myocardial Ischemia-Reperfusion Injury. Cells.

[90] Koltai M., Tosaki A., Hosford D., Braquet P. (1989). Ginkgolide B protects isolated hearts against arrhythmias induced by ischemia but not reperfusion. Eur. J. Pharmacol..

[91] Toldo S., Mauro A.G., Cutter Z., Abbate A. (2018). Inflammasome, pyroptosis, and cytokines in myocardial ischemia-reperfusion injury. Am. J. Physiol. Heart Circ. Physiol..

[92] Sinning C., Westermann D., Clemmensen P. (2017). Oxidative stress in ischemia and reperfusion: Current concepts, novel ideas and future perspectives. Biomark. Med..

[93] Morales C.R., Pedrozo Z., Lavandero S., Hill J.A. (2014). Oxidative stress and autophagy in cardiovascular homeostasis. Antioxid. Redox Signal..

[94] Chen-Scarabelli C., Agrawal P.R., Saravolatz L., Abuniat C., Scarabelli G., Stephanou A., Loomba L., Narula J., Scarabelli T.M., Knight R. (2014). The role and modulation of autophagy in experimental models of myocardial ischemia-reperfusion injury. J. Geriatr. Cardiol..

[95] Lazou A., Iliodromitis E.K., Cieslak D., Voskarides K., Mousikos S., Bofilis E., Kremastinos D.T. (2006). Ischemic but not mechanical preconditioning attenuates ischemia/reperfusion induced myocardial apoptosis in anaesthetized rabbits: The role of Bcl-2 family proteins and ERK1/2. Apoptosis.

[96] Yan H.F., Tuo Q.Z., Yin Q.Z., Lei P. (2020). The pathological role of ferroptosis in ischemia/reperfusion-related injury. Zool. Res..

[97] Luo G., Jian Z., Zhu Y., Zhu Y., Chen B., Ma R., Tang F., Xiao Y. (2019). Sirt1 promotes autophagy and inhibits apoptosis to protect cardiomyocytes from hypoxic stress. Int. J. Mol. Med..

[98] Zhong Z., Luo X.Y., Xiang P., Ji H.H., Wu X.D., Chong A.G., Hu X.Y., Cao X.L. (2023). MRTF-A alleviates myocardial ischemia reperfusion injury by inhibiting the inflammatory response and inducing autophagy. Mol. Cell. Biochem..

[99] Zweier J.L., Flaherty J.T., Weisfeldt M.L. (1987). Direct measurement of free radical generation following reperfusion of ischemic myocardium. Proc. Natl. Acad. Sci. USA.

[100] Blasig I.E., Ebert B., Hennig C., Pali T., Tosaki A. (1990). Inverse relationship between ESR spin trapping of oxyradicals and degree of functional recovery during myocardial reperfusion in isolated working rat heart. Cardiovasc. Res..

[101] Wu D., Ji H., Du W., Ren L., Qian G. (2022). Mitophagy alleviates ischemia/reperfusion-induced microvascular damage through improving mitochondrial quality control. Bioengineered.

[102] Ma L.L., Kong F.J., Dong Z., Xin K.Y., Wang X.X., Sun A.J., Zou Y.Z., Ge J.B. (2021). Hypertrophic Preconditioning Attenuates Myocardial Ischaemia-Reperfusion Injury by Modulating SIRT3-SOD2-mROS-Dependent Autophagy. Cell Prolif..

[103] Gao R., Lv C., Qu Y., Yang H., Hao C., Sun X., Hu X., Yang Y., Tang Y. (2023). Remote Ischemic Conditioning Mediates Cardio-protection After Myocardial Ischemia/Reperfusion Injury by Reducing 4-HNE Levels and Regulating Autophagy via the ALDH2/SIRT3/HIF1α Signaling Pathway. J. Cardiovasc. Transl. Res..

[104] Li J., Lu K., Zhang X., Wang T., Li Q., Yu X., Han W., Sun L. (2022). SIRT3-mediated mitochondrial autophagy in refeeding syndrome-related myocardial injury in sepsis rats. Ann. Transl. Med..

[105] Wang X., Xu Y., Li L., Lu W. (2021). Thrombin Aggravates Hypoxia/Reoxygenation Injury of Cardiomyocytes by Activating an Autophagy Pathway-Mediated by SIRT1. Med. Sci. Monit..

[106] Jia G., Hill M.A., Sowers J.R. (2018). Diabetic Cardiomyopathy: An Update of Mechanisms Contributing to This Clinical Entity. Circ. Res..

[107] Dewanjee S., Vallamkondu J., Kalra R.S., John A., Reddy P.H., Kandimalla R. (2021). Autophagy in the diabetic heart: A potential pharmacotherapeutic target in diabetic cardiomyopathy. Ageing Res. Rev..

[108] Kanamori H., Takemura G., Goto K., Tsujimoto A., Mikami A., Ogino A., Watanabe T., Morishita K., Okada H., Kawasaki M. (2015). Autophagic adaptations in diabetic cardiomyopathy differ between type 1 and type 2 diabetes. Autophagy.

[109] Yu W., Gao B., Li N., Wang J., Qiu C., Zhang G., Liu M., Zhang R., Li C., Ji G. (2017). Sirt3 deficiency exacerbates diabetic cardiac dysfunction: Role of Foxo3A-Parkin-mediated mitophagy. Biochim. Biophys. Acta. Mol. Basis Dis..

[110] Guo Z., Tuo H., Tang N., Liu F.Y., Ma S.Q., An P., Yang D., Wang M.Y., Fan D., Yang Z. (2022). Neuraminidase 1 deficiency attenuates cardiac dysfunction, oxidative stress, fibrosis, inflammatory via AMPK-SIRT3 pathway in diabetic cardiomyopathy mice. Int. J. Biol. Sci..

[111] Kawaguchi T., Takemura G., Kanamori H., Takeyama T., Watanabe T., Morishita K., Ogino A., Tsujimoto A., Goto K., Maruyama R. (2012). Prior starvation mitigates acute doxorubicin cardiotoxicity through restoration of autophagy in affected cardiomyocytes. Cardiovasc. Res..

[112] Sishi B.J., Loos B., van Rooyen J., Engelbrecht A.M. (2013). Autophagy upregulation promotes survival and attenuates doxorubicin-induced cardiotoxicity. Biochem. Pharmacol..

[113] Zheng D., Zhang Y., Zheng M., Cao T., Wang G., Zhang L., Ni R., Brockman J., Zhong H., Fan G.C. (2019). Nicotinamide riboside promotes autolysosome clearance in preventing doxorubicin-induced cardiotoxicity. Clin. Sci..

[114] Sun Z., Fang C., Xu S., Wang B., Li D., Liu X., Mi Y., Guo H., Jiang J. (2023). SIRT3 attenuates doxorubicin-induced cardiotoxicity by inhibiting NLRP3 inflammasome via autophagy. Biochem. Pharmacol..

[115] Shirakabe A., Zhai P., Ikeda Y., Saito T., Maejima Y., Hsu C.P., Nomura M., Egashira K., Levine B., Sadoshima J. (2016). Drp1-Dependent Mitochondrial Autophagy Plays a Protective Role Against Pressure Overload-Induced Mitochondrial Dysfunction and Heart Failure. Circulation.

[116] Zheng C.B., Gao W.C., Xie M., Li Z., Ma X., Song W., Luo D., Huang Y., Yang J., Zhang P. (2021). Ang II Promotes Cardiac Autophagy and Hypertrophy via Orai1/STIM1. Front. Pharmacol..

[117] Wang H.N., Li J.L., Xu T., Yao H.Q., Chen G.H., Hu J. (2020). Effects of Sirt3-autophagy and resveratrol activation on myocardial hypertrophy and energy metabolism. Mol. Med. Rep..

[118] Li J., Chen T., Xiao M., Li N., Wang S., Su H., Guo X., Liu H., Yan F., Yang Y. (2016). Mouse Sirt3 promotes autophagy in AngII-induced myocardial hypertrophy through the deacetylation of FoxO1. Oncotarget.

[119] Gao J., Wei T., Huang C., Sun M., Shen W. (2020). Sirtuin 3 governs autophagy-dependent glycolysis during Angiotensin II-induced endothelial-to-mesenchymal transition. FASEB J..

[120] Wei T., Huang G., Gao J., Huang C., Sun M., Wu J., Bu J., Shen W. (2017). Sirtuin 3 Deficiency Accelerates Hypertensive Cardiac Remodeling by Impairing Angiogenesis. J. Am. Heart Assoc..

[121] Zhong X.L., Miao H.J., Fang Z.M., Kuken B., Song H.Y., Zhong H., Lu Y., Liu S.M. (2015). The effect of SIRT1 gene polymorphisms on ambulatory blood pressure of hypertensive patients in the Kazakh population. Genet. Test. Mol. Biomark..

[122] Sakamoto J., Miura T., Shimamoto K., Horio Y. (2004). Predominant expression of Sir2alpha, an NAD-dependent histone deacetylase, in the embryonic mouse heart and brain. FEBS Lett..

[123] Lee D., Goldberg A.L. (2013). SIRT1 protein, by blocking the activities of transcription factors FoxO1 and FoxO3, inhibits muscle atrophy and promotes muscle growth. J. Biol. Chem..

[124] Li Z., Margariti A., Wu Y., Yang F., Hu J., Zhang L., Chen T. (2015). MicroRNA-199a induces differentiation of induced pluripotent stem cells into endothelial cells by targeting sirtuin 1. Mol. Med. Rep..

[125] Petrovski G., Gurusamy N., Das D.K. (2011). Resveratrol in cardiovascular health and disease. Ann. N. Y. Acad. Sci..

[126] Tanno M., Kuno A., Horio Y., Miura T. (2012). Emerging beneficial roles of sirtuins in heart failure. Basic Res. Cardiol..

[127] Chen M.L., Yi L., Jin X., Liang X.Y., Zhou Y., Zhang T., Xie Q., Zhou X., Chang H., Fu Y.J. (2013). Resveratrol attenuates vascular endothelial inflammation by inducing autophagy through the cAMP signaling pathway. Autophagy.

[128] Zhang Y., Cao X., Zhu W., Liu Z., Liu H., Zhou Y., Cao Y., Liu C., Xie Y. (2016). Resveratrol Enhances Autophagic Flux and Promotes Ox-LDL Degradation in HUVECs via Upregulation of SIRT1. Oxid. Med. Cell. Longev..

[129] Kanamori H., Takemura G., Goto K., Tsujimoto A., Ogino A., Takeyama T., Kawaguchi T., Watanabe T., Morishita K., Kawasaki M. (2013). Resveratrol reverses remodeling in hearts with large, old myocardial infarctions through enhanced autophagy-activating AMP kinase pathway. Am. J. Pathol..

[130] Zheng M., Bai Y., Sun X., Fu R., Liu L., Liu M., Li Z., Huang X. (2022). Resveratrol Reestablishes Mitochondrial Quality Control in Myocardial Ischemia/Reperfusion Injury through Sirt1/Sirt3-Mfn2-Parkin-PGC-1α Pathway. Molecules.

[131] Carrizzo A., Iside C., Nebbioso A., Carafa V., Damato A., Sciarretta S., Frati G., Di Nonno F., Valenti V., Ciccarelli M. (2022). SIRT1 pharmacological activation rescues vascular dysfunction and prevents thrombosis in MTHFR deficiency. Cell. Mol. Life Sci..

[132] Hu J., Zhang L., Yang Y., Guo Y., Fan Y., Zhang M., Man W., Gao E., Hu W., Reiter R.J. (2017). Melatonin alleviates postinfarction cardiac remodeling and dysfunction by inhibiting Mst1. J. Pineal Res..

[133] Yarmohammadi F., Barangi S., Aghaee-Bakhtiari S.H., Hosseinzadeh H., Moosavi Z., Reiter R.J., Hayes A.W., Mehri S., Karimi G. (2023). Melatonin ameliorates arsenic-induced cardiotoxicity through the regulation of the Sirt1/Nrf2 pathway in rats. BioFactors.

[134] Yu L.M., Dong X., Xue X.D., Xu S., Zhang X., Xu Y.L., Wang Z.S., Wang Y., Gao H., Liang Y.X. (2021). Melatonin attenuates diabetic cardiomyopathy and reduces myocardial vulnerability to ischemia-reperfusion injury by improving mitochondrial quality control: Role of SIRT6. J. Pineal Res..

[135] Pi Q.Z., Wang X.W., Jian Z.L., Chen D., Zhang C., Wu Q.C. (2021). Melatonin Alleviates Cardiac Dysfunction Via Increasing Sirt1-Mediated Beclin-1 Deacetylation and Autophagy During Sepsis. Inflammation.

[136] Qiao H., Ren H., Du H., Zhang M., Xiong X., Lv R. (2018). Liraglutide repairs the infarcted heart: The role of the SIRT1/Parkin/mitophagy pathway. Mol. Med. Rep..

[137] Yang M., Xi N., Gao M., Yu Y. (2022). Sitagliptin mitigates hypoxia/reoxygenation (H/R)-induced injury in cardiomyocytes by mediating sirtuin 3 (SIRT3) and autophagy. Bioengineered.

[138] Wang Y., Zhao R., Wu C., Liang X., He L., Wang L., Wang X. (2023). Activation of the sirtuin silent information regulator 1 pathway inhibits pathological myocardial remodeling. Front. Pharmacol..

[139] Zhang J., Cheng Y., Gu J., Wang S., Zhou S., Wang Y., Tan Y., Feng W., Fu Y., Mellen N. (2016). Fenofibrate increases cardiac autophagy via FGF21/SIRT1 and prevents fibrosis and inflammation in the hearts of Type 1 diabetic mice. Clin. Sci..

[140] Chang X., Zhang T., Liu D., Meng Q., Yan P., Luo D., Wang X., Zhou X. (2021). Puerarin Attenuates LPS-Induced Inflammatory Responses and Oxidative Stress Injury in Human Umbilical Vein Endothelial Cells through Mitochondrial Quality Control. Oxid. Med. Cell. Longev..

[141] Hui B., Hou X., Liu R., Liu X.H., Hu Z. (2021). Gypenoside inhibits ox-LDL uptake and foam cell formation through enhancing Sirt1-FOXO1 mediated autophagy flux restoration. Life Sci..

[142] Zhu Z., Li J., Zhang X. (2019). Salidroside protects against ox-LDL-induced endothelial injury by enhancing autophagy mediated by SIRT1-FoxO1 pathway. BMC Complement. Altern. Med..

[143] Jin X., Chen M., Yi L., Chang H., Zhang T., Wang L., Ma W., Peng X., Zhou Y., Mi M. (2014). Delphinidin-3-glucoside protects human umbilical vein endothelial cells against oxidized low-density lipoprotein-induced injury by autophagy upregulation via the AMPK/SIRT1 signaling pathway. Mol. Nutr. Food Res..

[144] Wang Y., Che J., Zhao H., Tang J., Shi G. (2019). Paeoniflorin attenuates oxidized low-density lipoprotein-induced apoptosis and adhesion molecule expression by autophagy enhancement in human umbilical vein endothelial cells. J. Cell. Biochem..

[145] Shi G., Liu D., Zhou B., Liu Y., Hao B., Yu S., Wu L., Wang M., Song Z., Wu C. (2020). Ginsenoside Rb1 Alleviates Oxidative Low-Density Lipoprotein-Induced Vascular Endothelium Senescence via the SIRT1/Beclin-1/Autophagy Axis. J. Cardiovasc. Pharmacol..

[146] Coelho A.R., Martins T.R., Couto R., Deus C., Pereira C.V., Simões R.F., Rizvanov A.A., Silva F., Cunha-Oliveira T., Oliveira P.J. (2017). Berberine-induced cardioprotection and Sirt3 modulation in doxorubicin-treated H9c2 cardiomyoblasts. Biochim. Biophys. Acta. Mol. Basis Dis..

[147] Mao S., Chen P., Li T., Guo L., Zhang M. (2018). Tongguan Capsule Mitigates Post-myocardial Infarction Remodeling by Promoting Autophagy and Inhibiting Apoptosis: Role of Sirt1. Front. Physiol..

[148] Sun X., Han Y., Dong C., Qu H., Yu Y., Ju J., Bai Y., Yang B. (2022). Daming capsule protects against myocardial infarction by promoting mitophagy via the SIRT1/AMPK signaling pathway. Biomed. Pharmacother..

[149] Xue Y., Fu W., Liu Y., Yu P., Sun M., Li X., Yu X., Sui D. (2020). Ginsenoside Rb2 alleviates myocardial ischemia/reperfusion injury in rats through SIRT1 activation. J. Food Sci..

[150] Hong Y.X., Wu W.Y., Song F., Wu C., Li G.R., Wang Y. (2021). Cardiac senescence is alleviated by the natural flavone acacetin via enhancing mitophagy. Aging.

[151] Guan S., Xin Y., Ding Y., Zhang Q., Han W. (2023). Ginsenoside Rg1 Protects against Cardiac Remodeling in Heart Failure via SIRT1/PINK1/Parkin-Mediated Mitophagy. Chem. Biodivers..

[152] Jiang Q., Lu M., Li J., Zhu Z. (2021). Ginkgolide B Protects Cardiomyocytes from Angiotensin II-Induced Hypertrophy via Regulation of Autophagy through SIRT1-FoxO1. Cardiovasc. Ther..

[153] Li W., Li X., Wang B., Chen Y., Xiao A., Zeng D., Ou D., Yan S., Li W., Zheng Q. (2016). ZLN005 protects cardiomyocytes against high glucose-induced cytotoxicity by promoting SIRT1 expression and autophagy. Exp. Cell Res..

[154] Zhang J., Cui J., Zhao F., Yang L., Xu X., Shi Y., Wei B. (2021). Cardioprotective effect of MLN4924 on ameliorating autophagic flux impairment in myocardial ischemia-reperfusion injury by Sirt1. Redox Biol..

[155] Zhu L., Duan W., Wu G., Zhang D., Wang L., Chen D., Chen Z., Yang B. (2020). Protective effect of hydrogen sulfide on endothelial cells through Sirt1-FoxO1-mediated autophagy. Ann. Transl. Med..

[156] Yang S., He J., Li X., Liu H., Zhao J., Liu M. (2018). Hydrogen attenuated oxidized low-density lipoprotein-induced inflammation through the stimulation of autophagy via sirtuin 1. Exp. Ther. Med..

[157] Fan L., Chen D., Wang J., Wu Y., Li D., Yu X. (2017). Sevoflurane Ameliorates Myocardial Cell Injury by Inducing Autophagy via the Deacetylation of LC3 by SIRT1. Anal. Cell. Pathol..

[158] Cong L., Gao Z., Zheng Y., Ye T., Wang Z., Wang P., Li M., Dong B., Yang W., Li Q. (2020). Electrical stimulation inhibits Val-boroPro-induced pyroptosis in THP-1 macrophages via sirtuin3 activation to promote autophagy and inhibit ROS generation. Aging.

[159] Wang P., Li M., Gao T., Fan J., Zhang D., Zhao Y., Zhao Y., Wang Y., Guo T., Gao X. (2023). Vascular Electrical Stimulation with Wireless, Battery-Free, and Fully Implantable Features Reduces Atherosclerotic Plaque Formation Through Sirt1-Mediated Autophagy. Small.

[160] Jiang Q., Hao R., Wang W., Gao H., Wang C. (2016). SIRT1/Atg5/autophagy are involved in the antiatherosclerosis effects of ursolic acid. Mol. Cell. Biochem..

[161] Wilson N., Kataura T., Korsgen M.E., Sun C., Sarkar S., Korolchuk V.I. (2023). The autophagy-NAD axis in longevity and disease. Trends Cell Biol..

[162] Saini R.K., Fulmor I.E., Parham C.S., Antonaccio M.J. (1986). Effects of SQ 26,533 on reperfusion arrhythmias, ST-segment elevation and on infarct size in anesthetized dogs. J. Pharmacol. Exp. Ther..

[163] Martorana P.A., Linz W., Göbel H., Petry P., Schölkens B.A. (1987). Effects of nicainoprol on reperfusion arrhythmia in the isolated working rat heart and on ischemia and reperfusion arrhythmia and myocardial infarct size in the anesthetized rat. Eur. J. Pharmacol..

[164] Hide E.J., Piper J., Thiemermann C. (1995). Endothelin-1-induced reduction of myocardial infarct size by activation of ATP-sensitive potassium channels in a rabbit model of myocardial ischaemia and reperfusion. Br. J. Pharmacol..

[165] Walsh S.K., Hepburn C.Y., Kane K.A., Wainwright C.L. (2010). Acute administration of cannabidiol in vivo suppresses ischaemia-induced cardiac arrhythmias and reduces infarct size when given at reperfusion. Br. J. Pharmacol..

[166] Qin X., Liu B., Gao F., Hu Y., Chen Z., Xu J., Zhang X. (2022). Gluconolactone Alleviates Myocardial Ischemia/Reperfusion Injury and Arrhythmias via Activating PKCε/Extracellular Signal-Regulated Kinase Signaling. Front. Physiol..

[167] Yaoita H., Ogawa K., Maehara K., Maruyama Y. (1998). Attenuation of ischemia/reperfusion injury in rats by a caspase inhibitor. Circulation.

[168] Kovacs P., Bak I., Szendrei L., Vecsernyes M., Varga E., Blasig I.E., Tosaki A. (2001). Non-specific caspase inhibition reduces infarct size and improves post-ischaemic recovery in isolated ischaemic/reperfused rat hearts. Naunyn Schmiedeberg’s Arch. Pharmacol..

